# The Response of a 16S Ribosomal RNA Gene Fragment Amplified Community to Lead, Zinc, and Copper Pollution in a Shanghai Field Trial

**DOI:** 10.3389/fmicb.2018.00366

**Published:** 2018-03-01

**Authors:** Shumeng Kou, Gilles Vincent, Emmanuel Gonzalez, Frederic E. Pitre, Michel Labrecque, Nicholas J. B. Brereton

**Affiliations:** ^1^Shanghai Chenshan Plant Science Research Center, Shanghai Chenshan Botanical Garden, Shanghai, China; ^2^Canadian Centre for Computational Genomics, McGill University and Genome Quebec Innovation Centre, Montréal, QC, Canada; ^3^Institut de Recherche en Biologie Végétale, Montreal Botanical Garden, Montréal, QC, Canada

**Keywords:** heavy metal contamination, bioremediation, 16S rRNA gene, soil bacteria, metagenomics

## Abstract

Industrial and agricultural activities have caused extensive metal contamination of land throughout China and across the globe. The pervasive nature of metal pollution can be harmful to human health and can potentially cause substantial negative impact to the biosphere. To investigate the impact of anthropogenic metal pollution found in high concentrations in industrial, agricultural, and urban environments, 16S ribosomal RNA gene amplicon sequencing was used to track change in the amplified microbial community after metal contamination in a large-scale field experiment in Shanghai. A total of 1,566 operational taxonomic units (OTUs) identified from 448,108 sequences gathered from 20 plots treated as controls or with lead, zinc, copper, or all three metals. Constrained Analysis of Principal Coordinates ordination did not separate control and lead treatment but could separate control/lead, zinc, copper, and three metal treatment. DESeq2 was applied to identify 93 significantly differentially abundant OTUs varying in 211 pairwise instances between the treatments. Differentially abundant OTUs representing genera or species belonging to the phyla Chloroflexi, Cyanobacteria, Firmicutes, Latescibacteria, and Planctomycetes were almost universally reduced in abundance due to zinc, copper, or three metal treatment; with three metal treatment abolishing the detection of some OTUs, such as *Leptolyngbya*, *Desmonostoc muscorum*, and *Microcoleus steenstrupii*. The greatest increases due to metal treatment were observed in Bacteroidetes, Actinobacteria, Chlamydiae, Nitrospirae, and Proteobacteria (α, β, δ, and γ); the most (relative) abundant being uncharacterized species within the genera *Methylobacillus*, *Solirubrobacter*, and *Ohtaekwangia*. Three metal treatment alone resulted in identification of 22 OTUs (genera or species) which were not detected in control soil, notably including *Yonghaparkia alkaliphila*, *Pedobacter steynii*, *Pseudolabrys taiwanensis*, *Methylophilus methylotrophus*, *Nitrosospira*, and *Lysobacter mobilis*. The capacity to track alterations of an amplified microbial community at high taxonomic resolution using modern bioinformatic approaches, as well as identifying where that resolution is lost for technical or biological reasons, provides an insight into the complexity of the microbial world resisting anthropogenic pollution. While functional assessment of uncharacterized organisms within environmental samples is technically challenging, an important step is observing those organisms able to tolerate extreme stress and to recognize the extent to which important amplifiable community members still require characterization.

## Introduction

Heavy metal contamination is a global environmental problem, but is a particular concern in China, in part due to recent, rapid industrial and economic development (Nriagu and Pacyna, 1988; Dong et al., 2001; Haiyan and Stuanes, 2003; Liu and Diamond, 2005; Wang and Zhang, 2006). Anthropogenic heavy metal pollution in China is thought to predominantly derive from mining, smelting, urban development, and industrial processing as well as sewage fertilization application (Cheng, 2003). Currently, around 10% of China’s arable lands, predominantly agricultural soils, are estimated as contaminated by heavy metals and that area is expected to expand due to the intensification of human activities in the coming decades (Teng et al., 2014; Zhang et al., 2015). The top four most metal polluted cities, based on an integrated pollution index (Cheng et al., 2014), have been ranked as Kunming, Chengdu, Nanning, and Shanghai; however, as could be anticipated, there is a substantial amount of geographical variation across China.

Lead, zinc, and copper contamination (amongst others) are at particularly high levels in Shanghai, making the city a suitable example for metal persistence within an urban environment. Cheng et al. (2014) measured an average of 103.6 mg Pb kg^-1^ in Shanghai topsoil but extremely localized more severe lead contamination was also present. As context, the Chinese national soil background level is 27 mg kg^-1^ (Chen et al., 1991), but values ranged across the city vary from 25.3 to 2,521 mg kg^-1^ [the well-regarded Canadian Environmental Quality Guidelines (CQG) maximum level for residential land is 140 mg kg^-1^ (CCME, 2014)]. The hotspots of extraordinarily high Pb pollution cover 108 km^2^ of the city (>140 mg kg^-1^ guideline) and are thought to be the result of long-term industrial activity. Importantly, the US Environmental Protection Agency (EPA) drinking water limits and health effect have been well reviewed in the context of remediation by Dixit et al. (2015) with lead concentrations above the limits of 15 ppm risking impaired development, reduced intelligence, short-term memory loss, disabilities in learning and coordination problems in children as well as the risk of cardiovascular disease more generally in the population. The average copper concentration of topsoil in Shanghai was measured at 63.8 mg Cu kg^-1^ soil and is thought to be the second most widespread copper contamination in metropolitan China, with >63 mg kg^-1^ concentrations measure over 28 km^2^ of the city (Cheng et al., 2014). This is above the CQG maximum for residential land levels of 63 mg kg^-1^ (CCME, 2014) (Chinese national soil background level 23 mg kg^-1^; Chen et al., 1991), but the pollution ranged from 21.2 to 420 mg kg^-1^. The potential for widespread health impact of copper contamination are high as levels above 1.3 ppm within water can result in brain and kidney damage, elevated levels result in liver cirrhosis and chronic anemia (Dixit et al., 2015). Extraordinarily high levels of zinc contamination are present in Shanghai which has the highest average topsoil content of 244 mg Zn kg^-1^ soil (ranging from 84 to 1,094 mg kg^-1^) in any city in China (Cheng et al., 2014) (the national background level being 74 mg kg^-1^; Chen et al., 1991). This is substantially above the CQG maximum levels for residential land of 200 mg kg^-1^ (CCME, 2014). Importantly, these high levels (>140 mg kg^-1^) cover almost the entirety of the Shanghai metropolitan area. Although is more widely considered non-toxic (so can biomagnify through the food chain; Wuana and Okieimen, 2011), long-term exposure to high zinc concentrations can cause headaches, nausea, stomach cramps, and diarrhea (Walsh et al., 1994).

The community of microbial life present in soil is essential for soil functions, such as nutrient cycling, decomposition, and mineralization, but also for the immobilization or removal of anthropogenic pollution (Giller et al., 1998; Dixit et al., 2015). While this functionality is the result of an operational community, the precise makeup of the community is still largely cryptic in nature. Traditional assessment of soil communities was contingent on successful culturing of microbial life isolated from soil and community-level physiological profiling (Hill et al., 2000); we know now that such assessment can underestimate the community, due partly to the difficulties in reproducing the complex habitats of nature *in vitro*. Even in light of modern molecular approaches for community assessment, such as ribosomal RNA gene amplification as an identification technique, it is still currently challenging to gauge this complex makeup as extensive bias and shortfalls prevent comprehensive community identification in its entirety (Sun et al., 2013; Vetrovsky and Baldrian, 2013; Nguyen et al., 2016). Substantial inroads have and are being made in these technologies, with culturing techniques improving the capability to simulate natural environments (in line with discoveries of such complexity in nature; Stewart, 2012) and the error rates of longer read sequencing technology are being reduced, moving toward sequences which can be used more confidently as an identification marker at a phylogenetically relevant length within high complexity metagenomic samples (D’Amore et al., 2016).

Heavy metal contamination should impact the diversity of microbes in a soil if individual members of that community are sufficiently susceptible (lacking resistance) to high metal concentrations before the wider community functionality can reduce and ultimately recover from such stress (resilience) (Griffiths and Philippot, 2013). The current uncertainties associated with microbial community assessment have, however, given rise to sometimes contrasting observations as to the impact of anthropogenic pollution to soil microbial diversity and functionality. For example, several studies have outlined a lack of microbial community resistance to metal contamination, memorably that of Gans et al. (2005), who suggested a 99.9% reduction in soil microbial diversity due to the application of heavy metal contaminated sludge, while others have demonstrated extensive community resistance or resilience to metal contamination (Azarbad et al., 2016). There are examples of most phyla [represented as operational taxonomic units (OTUs) in 16S rRNA studies] having been observed in metal contaminated conditions, which could be expected as only a single member species needs to be tolerant to meet this phyla level criteria. Examples of high taxon groupings having at least some generalized published precedence of tolerance to anthropogenic pollution include, but are not limited to: Archaea (Voica et al., 2016), Eukaryota (Tyler, 1990; Pulford and Watson, 2003; Masmoudi et al., 2013), Cyanobacteria (Fiore and Trevors, 1994), Chloroflexi (Yin et al., 2015), Firmicutes (López-Archilla et al., 2004), Planctomycetes (Raji et al., 2008), Latescibacteria (Hernandez-Raquet et al., 2006), Actinobacteria (Zhang et al., 2007a; Lejon et al., 2008), Proteobacteria (Zhu et al., 2013), Nitrospirae (Dionisi et al., 2002; Liu et al., 2014), Acidobacteria (Berg et al., 2012b), Bacteroidetes (Ellis et al., 2003; Rastogi et al., 2009), and Chlamydiae (Zhang et al., 2007b; Chen et al., 2014). Importantly, this strongly implies the necessity to resolve microbial community description below the level of phylum (to lower levels of taxonomy).

While 16S rRNA gene fragment sequencing cannot provide confident assessment of the entire microbial community, as universal primers do not exist (Rosselli et al., 2016; Martinez-Porchas et al., 2017) (hyper-conserved 16S rRNA regions are sometimes not conserved, much like hyper-variable regions are sometimes conserved), the relative abundance of microbes whose 16S rRNA gene target region is amplified (the amplified community) with a given pair of primers can be compared within a sample, and that relationship of abundance can therefore be compared between experimental treatments. As such, relative changes in the amplified community between treatments are well-suited for comparison by differential abundance (DEseq2; Love M. et al., 2014); similar to differential expression analysis commonly used in transcriptomic studies). In this research, we investigate the individual effect of lead, zinc, and copper as well as a mix of the three metals on the diversity and relative abundance of the amplified microbial community identified using 16S rRNA gene amplicon sequencing.

## Materials and Methods

### Site, Soil Properties, and Experimental Design

**FIGURE 1:**
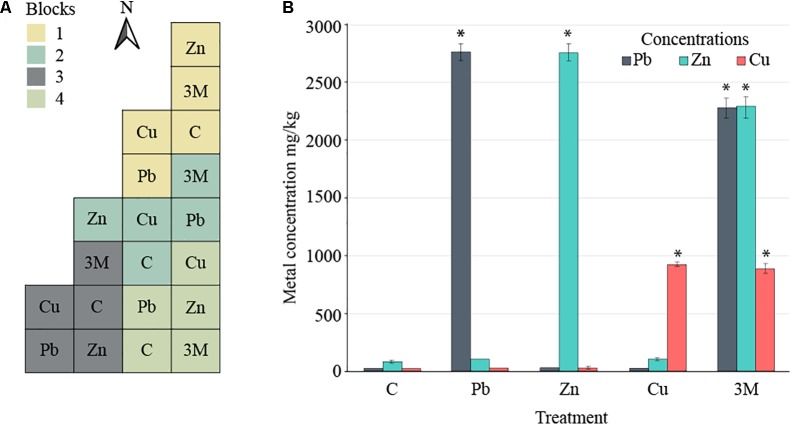
Field design. **(A)** Split-plot experimental design comprising four blocks divided in five 10 m × 10 m plots, each corresponding to one of the five treatments: control soil (C) or soil spiked with Cu, Pb, Zn, or a mix of all three metals (3M). **(B)** The mean concentration of spiked metals across each of the five treatments at the time of sampling for DNA extraction and 16S rRNA gene amplicon analysis (error bars = standard error of four blocks per treatment). Asterisk “^∗^” represents statistical difference from control soil for a given metal (*t*-test, *p* < 0.05).

The site was located at the Shanghai Chenshan Botanical Garden (N31°04′39″, E121°11′12″) in China. Soil pH and electrical conductivity were determined using a Hach pH meter (Hach Company, United States) and electrical conductivity instrument (Leici Company, China) on the supernatant of 1:5 soil and water mixtures. Soil properties were determined as outlined in Yan et al. (2009); briefly, a SmartChem 200 flow-injection autoanalyzer was used to assess total N concentration (Kjeldahl) after digested with H_2_SO_4_ in glass tubes. Total C concentration of K_2_SO_4_-extracted solutions were measured using an automated Total Organic Carbon Analyzer (Shimadzu, TOC-Vcph, Japan). Soil organic matter (SOM) content using the oil bath—K_2_CrO_4_ titration method. Soil bulk density (BD) cores were also collected (using a 100 cm^3^ cylinder) from each plot for determination of BD, soil water content, and general porosity after being weighed before and after oven drying at 105°C for 24 h. The split-plot experimental design comprised four blocks divided in five 10 m × 10 m plots, in which the five treatments were randomly applied (**Figure 1A**). These treatments included spiking the soil with salts of different metals (Pb, Zn, and Cu), a treatment containing a mix of the three metals, and a control treatment without contamination. CuCl_2_ (34.22 kg plot^-1^), PbCl_2_ (65.35 kg plot^-1^), and ZnCl_2_ (101.83 kg plot^-1^) powders were applied as a spike to soil for each treatment in autumn 2014, mechanically incorporated into the first 25–30 cm, and left to equilibrate before samples were taken in autumn 2015 to assess metal content (**Table 1**). Four bulk soil samples (30 cm topsoil) taken in 2015 were homogenized and pooled for each plot. Soil samples were taken in a similar manner in autumn 2016 and soil was again assessed for metal content. Soil samples were air-dried before sieving (2-mm mesh) prior to Pb, Zn, and Cu concentration quantification using HNO_3_ digestion for 5 h at 120°C, before inductively coupled plasma mass spectrometry. Each soil sample was assessed in duplicate (technical replicates), with method blanks and with reference material from the Chinese Academy of Measurement Sciences with recovery yields of all peaks ranging from 90 to 105%.

**Table 1:** Soil physiochemical characteristics.

Soil parameters	Control	Copper treatment	Zinc treatment	Lead treatment	Three metal treatment
pH	8.1 ± 0.1a	7.6 ± 0.1b	7.2 ± 0.3c	6.9 ± 0.2c	6.0 ± 0.2d
EC (μS cm^-1^)	205.6 ± 22.6d	544.3 ± 79.3c	711.8 ± 72.4c	980.0 ± 110.5b	2607.3 ± 198.7a
SOM (mg g^-1^)	16.5 ± 3.2a	15.6 ± 1.1a	17.1 ± 5.1a	17.5 ± 1.11a	17.3 ± 6.1a
TOC (mg g^-1^)	10.0 ± 1.6a	9.2 ± 0.6a	10.1 ± 3.0a	9.3 ± 2.8a	10.9 ± 4.0a
BD (mg m^-3^)	1.4 ± 0.1a	1.4 ± 0.1a	1.4 ± 0.1a	1.4 ± 0.2a	1.5 ± 0.1a
Nitrogen (mg g^-1^)	0.86 ± 0.049a	0.77 ± 0.004a	0.79 ± 0.071a	0.73 ± 0.058a	1.04 ± 0.083a
Phosphorus (mg g^-1^)	0.010 ± 0.005b	0.025 ± 0.005a	0.022 ± 0.010ab	0.022 ± 0.010ab	0.017 ± 0.007ab
WC (%)	27.5 ± 5.6a	28.0 ± 7.7a	26.0 ± 2.5a	32.6 ± 4.5a	26.3 ± 4.1a
General porosity (%)	45.3 ± 3.9a	46.1 ± 7.4a	43.5 ± 2.1a	48.9 ± 11.6a	42.6 ± 2.7a
CuCl_2_ (kg plot^-1^)	0	34.22	0	0	34.22
ZnCl_2_ (kg plot^-1^)	0	0	101.83	0	101.83
PbCl_2_ (kg plot^-1^)	0	0	0	65.35	65.35
Cu content (mg kg^-1^)	89.3 ± 11.4b	1978.4 ± 175a	65.4 ± 15b	78.2 ± 8.6b	1749.1 ± 649.1a
Zn content (mg kg^-1^)	75.5 ± 4.7c	81.3 ± 12.1c	3206.3 ± 423.8a	71 ± 1.8c	2610.5 ± 679.5b
Pb content (mg kg^-1^)	20.0 ± 7c	24.5 ± 11.1c	21.6 ± 6.8c	4067.5 ± 915.8a	1745.8 ± 254.2b


*The mean of four plots per treatment and standard deviation is shown. The lowercase letters indicate least significant difference (LSD) *post hoc* test, *p* < 0.05.*

### DNA Extraction, PCR Amplification, and 454 Pyrosequencing

The bulk soil samples taken in November 2016 were also used for DNA extraction and 16S ribosomal RNA gene amplicon sequencing. Genomic DNA was extracted using OMEGA Soil DNA Kit (D5625-01), according to the manufacture’s protocol. The quantity and quality of the extracted DNA were examined using an Eppendorf RS232G UV-Vis spectrophotometer. The V4-V5 region of 16S rRNA genes were PCR amplified from bacteria compatible with the primers 515F (GTGCCAGCMGCCGCGGTAA; Tringe and Hugenholtz, 2008) and 907R (CCGTCAATTCMTTTGAGTTT; Sogin et al., 2006) following the conditions: initial denaturation 98°C for 2 min, denaturation 98°C for 15 s, annealing 55°C for 30 s, extension 72°C for 30 s, final extension 72°C for 5 min, 10°C hold, followed by 25–30 cycles. The PCR products were purified using an AxyPrep DNA Gel Extraction Kit according to the manufacture’s protocol before pyrosequencing on the Roche 454 GS-FLX Titanium sequencer (Roche 454 Life Sciences, Branford, CT, United States) at Personalbio (Shanghai Personal Biotechnology, Co., Ltd., Shanghai, China).

### Sequencing Analysis

**FIGURE 2:**
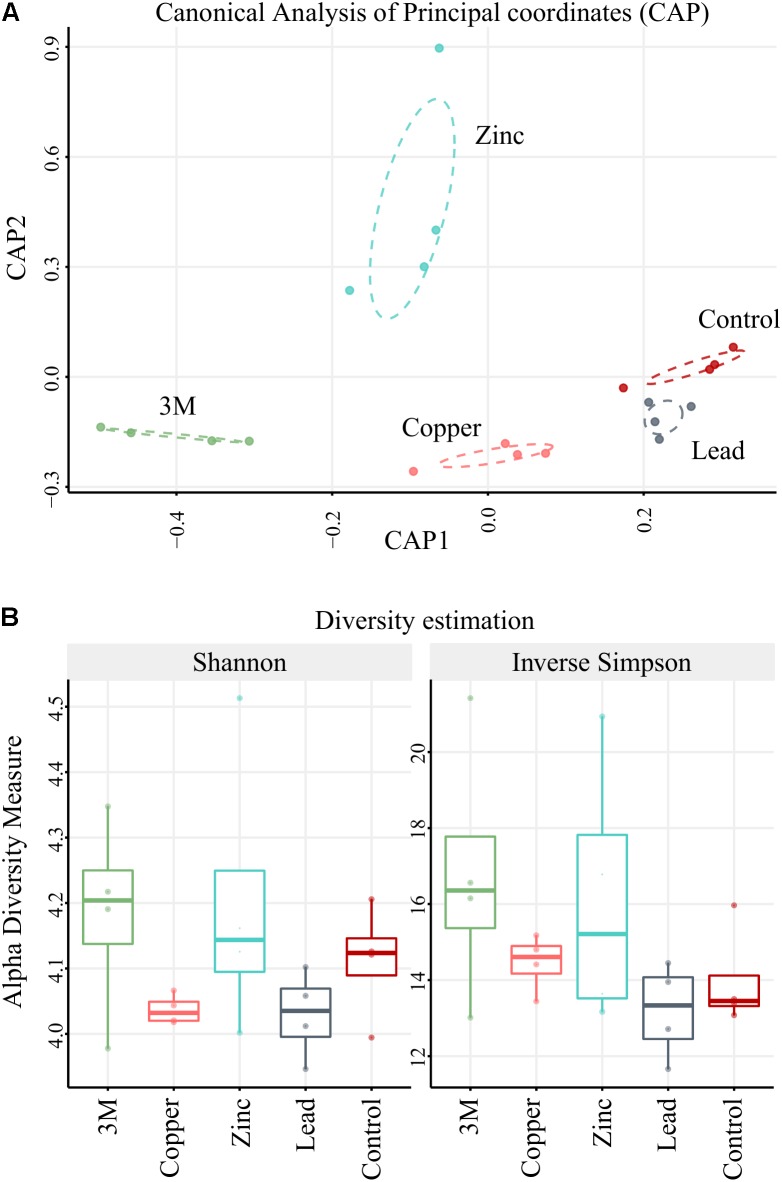
CAP and diversity. **(A)** Ordination plot using Canonical Analysis of Principal coordinates model (CAP) based on Bray–Curtis distances. **(B)** Compared alpha-diversity box plots for each treatment and control based on two diversity estimators: Shannon and Inverse Simpson indices. *n* = 4 replicate blocks per treatment.

The Quantitative Insights Into Microbial Ecology (QIIME; Caporaso et al., 2010) suite of analysis tools were used to filter and analyze the sequence data. A total of 448,102 sequences were obtained across all 20 plots after removal of the index and primer sequences, quality control filtering and rarefaction. All amplicons were annotated using blastn on NCBI database where all hits with an e-value below a threshold of 10^-10^ were retained for each sequence, allowing a large base of potential hits for each query sequence. All potential annotation hits were then filtered at a strict criteria of ≥99% identity and ≥99% alignment coverage on any query sequence. For each amplicon, “unknowns” or “uncharacterized” labels were removed when possible and the hit with the highest bit score was selected as annotation. For ambiguous calls (identical bit score), the lowest shared taxonomic level of all potential hits was used. Amplicon annotation was then considered as OTUs (for readability, we borrow the commonly used OTU for readability; these do not represent consensus sequences or clustered amplicons but annotation-binned sequences). Amplicons without annotation (<99% identity and coverage to known NCBI nt sequences) were binned to OTUs using the more forgiving criteria of ≥98% identity and ≥98% alignment coverage. No new OTU construction was permitted in this step (which is used to increase capture of artificial amplicons produced by sequencing error as opposed to establishing less confident OTU). Lastly, in an effort to reduce the typical sparsity encountered in rRNA OTU tables, we flagged OTU that were present in fewer than four biological replicates and those with a high total count concentrated in a single sample (OTU maximum count in a sample: OTU total count of all samples <0.75, **Supplementary File 1**). The final 1,566 OTU table count showed similar depth within all samples (**Supplementary File 1**). Constrained Analysis of Principal Coordinates (CAP; **Figure 2A**) ordination was performed based on Bray–Curtis ecological distances using Phyloseq package (McMurdie and Holmes, 2013). Dispersion ellipses were drawn using veganCovEllipse function from Vegan package (Oksanen et al., 2007) in R (R Development Core Team, 2014). Alpha diversity was measured using six different indices [Shannon, inverse Simpson (**Figure 2B**), Observed, Chao1, Simpson, and Fisher (**Supplementary File 1**) within Phyloseq package (McMurdie and Holmes, 2013)]. Alpha-diversity was compared between the different groups of samples using a *t*-test. Of 1,566 constructed OTU, only 642 were retained as >10 total normalized counts across all samples to minimize interference during the standard size factor estimator in the subsequent DESeq2.

To characterize statistically significant differentially abundant OTUs between groups of samples, parametric models developed in transcriptomics have been shown to perform well when applied to the pitfalls of microbiome biomarker data (uneven library sizes, sparsity, sample representatively; Weiss et al., 2017). The univariate DESeq2 method was used to identify differentially abundant OTUs (Love M.I. et al., 2014; Thorsen et al., 2016) with a *p*-value and false discovery rate cut-off (Benjamini–Hochberg) <0.05 applied after the statistical tests. Rlog data transformation was used (blind = TRUE) as it offers a safer curve of the square root of variance over the mean (**Supplementary File 1**). Differential abundance is not implicit if an OTU has counts in one condition but is not detected in another within a given comparison due to the requirement of abundance (presence) to be above DESeq2 significance thresholds (population noise), but is more likely. Similarly, the absence of any counts for an OTU within plots of soil from a single condition does not distinguish between absence of the bacteria and very low abundance (below detection, particularly relevant due to rarefaction; Weiss et al., 2017), so is considered only as potential absence throughout the discussion. Any amplicon’s utility to distinguish particular species within a genus, or even genera within a family, is variable and it can be useful to iteratively inform bioinformatics decision-making of the specific biology of the environment as well as to allow for acknowledgment of that variability through comprehensive OTU investigation. OTU unpacking analyses separated sequences into sub-OTUs when no specific annotation accession hit was shared between an OTU’s constituent sequences. For example, the same annotation label (i.e., so common OTU) but with different accession numbers would give rise to two sub-OTUs. The majority of 16S rRNA gene annotation in databases (NCBI nt, SILVA, etc.) derives from *in silico* sequence comparison, so has the potential to be distant from sequencing-independent characterized bacteria; therefore, for comparison, phylogenetic trees were built by phylum including spikes of the closest isolated/cultured relatives (Ellis et al., 2003; Zhang et al., 2012) based on the aligned 16S rRNA gene fragment from the 16S archaea and bacteria NCBI database. Unique identifiers (accession details) for every spike sequence are available in **Supplementary File 2**. Phylogenetic trees used Jukes–Cantor model, through a preference for simplicity (of substitution model) given the multiple potential levels of selection across the entirety of 16S rRNA gene (Řeháková et al., 2014; Ziesemer et al., 2015), and neighbor-joining method (La Rosa et al., 2013). Newick phylogenetic trees were visualized using iTOL (Interactive Tree of Life).

## Results and Discussion

### Amplified Community Structure and Differentially Abundant OTUs

Two years after the application of metals to the soil, in August 2016 (just before sampling for DNA extraction), the Pb-treated soil had 2751.57 mg Pb kg^-1^ soil [standard error (SE) 74.69], Zn-treated soil averaged 2755.19 mg Zn kg^-1^ soil (SE 73.89), Cu-treated soil averaged 925.73 mg Cu kg^-1^ soil (SE 14.42) and three metal-treated soils averaged 2270.17 (SE 90.51), 2281.11 (SE 93.24), and 885.36 (SE 43.76) mg of Pb, Zn, and Cu kg^-1^ soil, respectively (**Figure 1B**). The substantial changes in metal concentrations between autumn 2015 and autumn 2016 were a 14% reduction in lead measured from lead-treated soils as well as a 13–14 and 49–53% reduction in zinc and copper measure from their individual treatments or three metal-treated soils. Increases were observed in lead concentrations (30%) within three metal-treated soils which could be either due to technical error or a change in soil localization over time.

The 448,102 high-quality amplicon sequences had an average length of 394 bp from all 20 soil samples (5 treatments × 4 replicate 100 m^2^ plots). A total of 1,566 OTUs were constructed based on >99% blastn identity and >99% coverage of hits in the NCBI nt database, with a bias for the lowest taxon hit available at that criteria. A total of 213,534 amplicon sequences were binned into high confidence OTUs in this first round (47.65% of amplicon sequences present after rarefaction). A second round gathered 49,985 un-binned sequences into established OTUs at >98% identity so that the total number of sequences gathered was 263,519 (58.81% of sequences present after rarefaction). To further analyze CAP ordination (**Figure 2A**), permutational multivariate analysis of variance showed between group OTU count variation to be significantly greater than the variation within the groups (Pr < 0.001), indicating there were differences in the amplified community makeup between treatments. OTU diversity representation with diversity indices Shannon and inverse Simpson (**Figure 2B**) identified no significant difference (*t*-test); the high *p*-values could be due to small group size comparisons (**Supplementary File 1**). The majority of the amplified community did, however, demonstrate incredibly high resilience to metal contamination, as 86% of OTUs (549/642 OTU) were not differentially abundant due to a treatment. This suggests that the amplified microbial community, in the main, could have either returned or remained as similar in structure to untreated control plots 2 years after metal treatment. Although the diversity of the amplified microbial community was relatively stable, 93 OTUs were identified using DESeq2 as statistically altered in abundance (differentially abundant) with respect to treatment.

**FIGURE 3:**
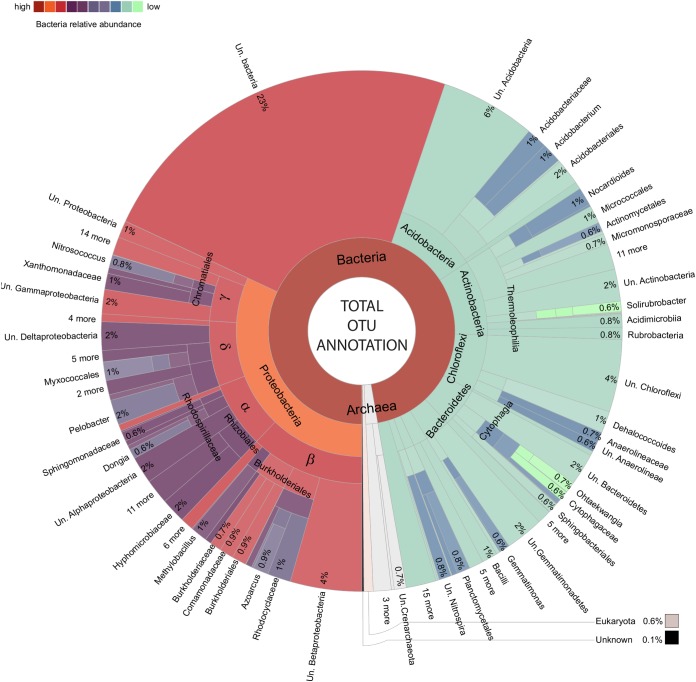
Total amplified community makeup. Krona chart (Ondov et al., 2011) presenting an overview of OTUs.

An important element in realizing the benefits of modern environmental sequencing for microbial community assessment is the recognition of our current limitations both technically and in terms of biological understanding. The very high (taxonomically) resolved inventory of the soil microbiota tolerating metal contamination are carefully outlined as the *amplified* 16S rRNA gene community in order to prevent a reiteration of the mistakes of the past, in particular the belief that laboratory culturing could always characterize the entirety of a community within a given unknown biological sample. The utility of amplicons to distinguish species is dependent on the variation in 16S rRNA gene copy number within a strain/species (Farrelly et al., 1995; Vetrovsky and Baldrian, 2013) and the variation in 16S rRNA gene/s from a closely related species (Sun et al., 2013; Vetrovsky and Baldrian, 2013) as well as sometimes hindered by taxonomic resolution or the number of characterized species within a given database (which likely include only a small minority of those existing in the biosphere; Locey and Lennon, 2016; Balvočiūtė and Huson, 2017). The construction of “OTUs” (consensus sequences derived from similarity clusters) is used in 16S rRNA gene sequencing (to combat this inherent shortfall in the utility of some amplicons as unique species level identification markers (alongside sequencing error rates), however, this can sometimes obscure biologically useful complexity which is present and accessible within data (**Figure 3**). Here, the relative utility as an identification marker is considered on an amplicon by amplicon basis by unpacking each OTU into distinct constituent sequences (sub-OTUs). These sub-OTUs are then be compared against the context of aligned 16S rRNA gene sequence from well-characterized bacteria represented in NCBI’s curated 16S bacteria and Archaea rRNA gene database, where the majority of records are from cultured/isolated bacteria or are manually curated (**Figures 5**–**9** and **Supplementary File 3**). This allows for the identification of known species which cannot be separated using this amplicon (where the amplified sequence is conserved between species), valuable in establishing confidence, or lack thereof, in the species present within the community. All differentially abundant OTUs with annotation at genera or species level are discussed in the context of any recognized putative lifestyle or ecological function.

In terms of pairwise comparison, none of the 93 differentially abundant OTUs identified as significantly responsive to metal treatment were DA when comparing control with lead-treated soil, 17 were differentially abundant comparing zinc with control, 8 comparing copper with control, 68 comparing all three metals with control, 15 comparing zinc with lead, 2 comparing copper with lead, 60 comparing all three metals with lead, 6 comparing copper with zinc, 12 comparing all three metals with zinc, and 23 comparing all three metals copper. The lack of any impact of lead treatment upon the diversity or abundance of soil bacteria contrasts previous laboratory experiments by Sobolev and Begonia (2008) who found even very small concentrations (1 ppm) negatively influenced soil diversity. Lead was the only non-bioessential metal applied to soil; although lead is considered extremely toxic microorganisms even at low concentrations (Naik and Dubey, 2013), high microbial diversity (similarity to control soil; **Figure 2B**) has been previously observed when using the 16S rRNA gene as an identification marker (Gołȩbiewski et al., 2014) and may be explained by immobilization due to the adequate soil phosphate levels (Park et al., 2011a,b) (**Table 1**) or longer-term microbial community resilience mechanisms.

**FIGURE 4:**
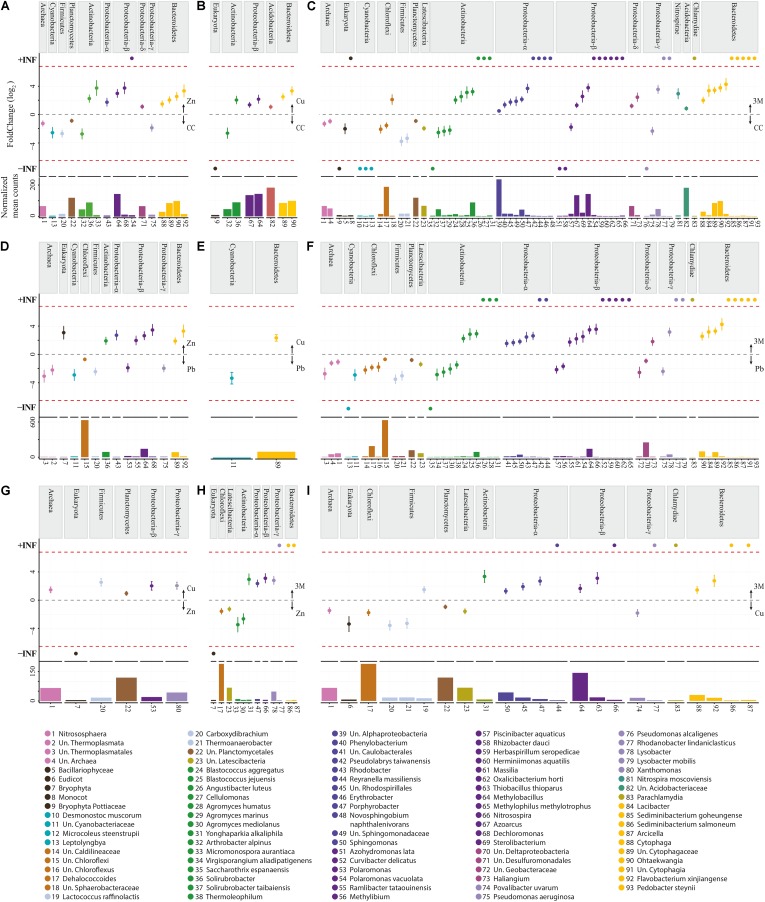
Differentially expressed OTUs: abundance and fold change. **(A)** Control (CC) vs Zn; **(B)** CC vs Cu; **(C)** CC vs three metals (3M); **(D)** Pb vs Zn; **(E)** Pb vs Cu; **(F)** Pb vs 3M; **(G)** Zn vs Cu; **(H)** Zn vs 3M; **(I)** Cu vs 3M. No OTUs were identified as differentially abundant between CC and Pb treatments. Fold change (FC Log_2_) denotes relative differences in abundance between treatments (directionality is indicated with arrows on the right of each graph). +/- INF indicates “infinite” fold change, where an OTU had counts in only a single condition. Normalized mean counts originate from DESeq2 basemean output (note: *y*-axis max is not constant). Species are grouped by phylum per comparison as well as using color.

These differentially abundant OTUs include 63 instances where an OTU presence was either abolished by a metal treatment or newly detected to the amplified community due to a metal treatment (absent from soil of a comparative treatment) (**Supplementary File 2**). The majority of newly detected OTUs (50) were identified in soils treated with all three metals and were predominately Proteobacteria or Bacteroidetes. As opposed to the original community members tolerant to the metal contamination, these OTUs potentially represent metal-resistant extremophiles with particular selective advantage within these stressful conditions. The most substantial treatment effect, or greatest number of putative microbial populations altered in abundance within the amplified community, was due to application of all three metals, with zinc and copper treatments both having an intermediate effect (this is illustrated well using CAP ordination, **Figure 2A**). The differentially abundant OTUs are considered in separate groups of Archaea, Eukaryota, and into 11 bacterial phyla, with Proteobacteria being further separated into class as having a number distinct differentially abundant OTUs in Alphaproteobacteria (eight OTUs), Betaproteobacteria (eight OTUs), Deltaproteobacteria (four OTUs), and Gammaproteobacteria (four OTUs) (**Supplementary File 2**). The significant increase or decrease in OTU abundance was similar within phyla, contrary to an expectation that species level resolution of the amplified community would reveal that each phyla encompasses a wealth of metal tolerant and metal intolerant species (making generalized functional interpretation problematic at the phylum level). The clear patterning in significant differential abundance of OTU at a phylum level is potentially a reassuring finding, given that the overwhelming majority of contemporary microbial community assessment studies are performed at phylum or class taxon level only. Almost all Archaea, Eukaryota, Cyanobacteria, Chloroflexi, Firmicutes, Planctomycetes, and Latescibacteria OTUs were significantly reduced in abundance due to zinc, copper, and/or three metal treatments (when compared to control or lead treatment), whereas Actinobacteria, Proteobacteria, Nitrospirae, Acidobacteria, Bacteroidetes, and Chlamydiae were generally increased in abundance (**Figure 4**).

#### Archaea and Eukaryota

**FIGURE 5:**
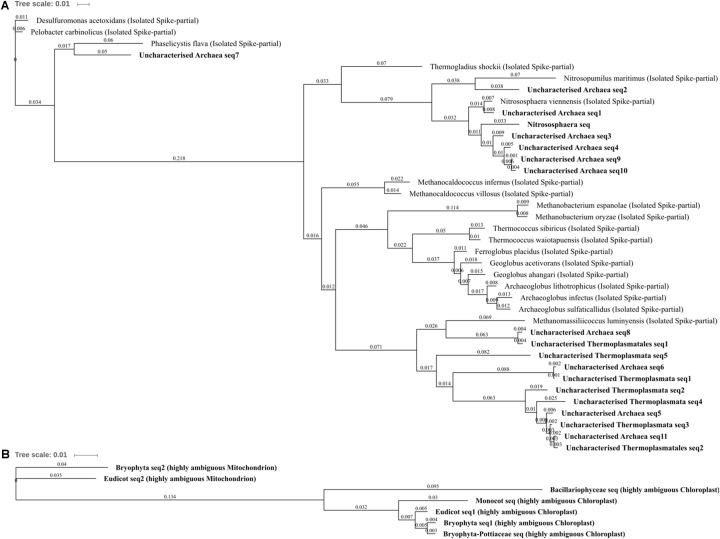
Archaea and Eukaryota phylogenetic tree. **(A)** Archaea, **(B)** Eukaryota. Jukes–Cantor model with neighbor-joining and >50% support threshold (1,000 replications). Sub-OTUs (distinct amplicons) are highlighted in bold with spike (into the tree) sequences included as the most similar hits from the NCBI bacteria and archaea 16S rRNA gene database. All accession numbers and sequences (sub-OTUs and tree spikes) are provided in **Supplementary File 2**. The value “0.01” on the scale bar represents 1 substitution in 100 bp.

The differentially abundant archaeal OTUs: uncharacterized Thermoplasmata, uncharacterized Thermoplasmatales, *Nitrososphaera*, and uncharacterized Archaea, were generally poorly characterized and are indicative of the need for more extensive Archaeal 16S rRNA gene research as few characterized (reference) species were highly similar to those present within this soil (which were common/abundant uncharacterized sequences in the NCBI nt database). The most abundant archaeal OTU was *Nitrososphaera* (**Figure 5**; 0.67% total normalized counts in control soil) which comprised only a single sub-OTU most similar to the isolated *Nitrososphaera viennensis* (Stieglmeier et al., 2014): a mesophilic and neutrophilic aerobe being the first cultivated (from soil) ammonia oxidizer. *Nitrososphaera* were reduced in three metal- and zinc-treated soil when compared with control as well as in three metal treatment compared with lead- and copper-treated soil, but by a relatively small degree from -1.11 to -1.43 log_2_FC. *Nitrososphaera* was less impacted by copper-treated soil, which was not significantly altered in comparison to control and in higher abundance when compared to zinc (1.35 log_2_FC; **Figure 4G**). All of the sub-OTUs within the uncharacterized Thermoplasmata (and uncharacterized Thermoplasmatales), a class comprised of species distinct from *Nitrososphaera* in being thermophilic and acidophilic, were all most similar (blastn identity 83–87%) to the isolated *Methanomassiliicoccus luminyensis. M. luminyensis*, while often found in the human gut, has recently been proposed as a member of a seventh order of methanogens and widely observed in hydrothermal vents and soils (Paul et al., 2012). A clear pattern of reduced abundance of all the Archaea species due to zinc and copper treatments, similar to that observed in zinc and copper contaminated sewage sludge by Sandaa et al. (1999), strongly suggests these species are either metal intolerant or less competitive in the metal-altered microbial community.

For each of the major Eukaryota taxa, the amplicon was highly conserved, but was distinct between Eudicots, Monocots, Bryophytes, and Bacillariophyceae (diatoms). Although these primers do not amplify sequences which can be used to separate Eukaryota genera, it is important to recognize all amplicons where possible in a sample as retention of information is vital to allow for the unexpected and to help identify potentially confounding biology. Although studies such as that by López-Archilla et al. (2001) in Spain have identified Bacillariophyceae OTU in copper and zinc contaminated conditions, here they were abolished (not detected) by copper and three metal treatments when compared to control. The very similar (>98% blastn identity) ambiguous Bryophyta and Bryophyta-Pottiaceae OTUs were also abolished entirely by copper and three metal treatments compared to control and zinc treatments indicating a potentially extreme susceptibility to copper but tolerance to zinc treatment. This zinc tolerance was further affirmed as the Bryophyta OTU was in significantly and substantially higher abundance in zinc- than lead-treated soils (3.07 log_2_FC; **Figure 4D**). Several Bryophytes are known to be tolerant of high concentrations of lead, zinc, and copper (Tyler, 1990) and many Pottiaceae species abundant across North America and China, such as *Scopelophila cataractae* (“copper moss” or “metal moss”), are and well-recognized as lead, zinc, and copper tolerant (Shaw, 1987). The ambiguous monocot OTU was in greater abundance in three metal-treated soils and also absent from control soil. There are several zinc tolerant mosses and broadly metal tolerant or hyperaccumulating monocots; however, due to the poor taxonomic resolution, little further can be speculated upon using the 16S rRNA gene alone. What can be further garnered when OTUs were unpacked was that diatom and monocot sequences could easily (due to the extensive species conservation) be identified as chloroplastic in origin. Further to this, Bryophyta and Eudicot OTUs were comprised of only two distinct sub-OTUs, both of which corresponded to chloroplast and mitochondrial 16S rRNA gene and were present in abundance ratios of approximately 3:1, respectively. This could suggest variation in gene copy number or organelle number per cell of a predominant tissue, of a species present or of a diversity of species in the soil.

#### Cyanobacteria

Four cyanobacteria OTUs were differentially abundant because of metal treatment to soil: uncharacterized Cyanobacteriaceae, *Leptolyngbya*, *Desmonostoc muscorum*, and *Microcoleus steenstrupii*. Uncharacterized Cyanobacteriaceae was reduced in abundance in zinc-, copper-, and three metal-treated soil when compared to lead treatment (**Figures 4D–F**). *Leptolyngbya* presence was abolished in soil treated with three metals and zinc as was *D. muscorum* and *M. steenstrupii* in soil treated with three metals. The *Leptolyngbya* OTU contained seven distinct sub-OTUs, with the most similar well-characterized isolated species (present in the NCBI bacteria and archaea 16S rRNA gene database) sharing <92% blastn identity (**Supplementary File 3**). The genus contains over 140 species as well as unclear taxa which include a number of *Oscillatoria* and *Phormidium* species, so it is unsurprising that the most similar isolated species included *Oscillatoria acuminate* and *Phormidium etoshii*. The *D. muscorum* OTU was comprised of four sub-OTUs most similar to three *Cylindrospermum* species (*C. licheniforme*, *C. pellucidum*, and *C. marchicum*) as well as *Nostoc punctiforme* (98% blastn identity to *D. muscorum* seq4). Hudek et al. (2009) found *N. punctiforme* had some tolerance to zinc and contains a metallothionein (SmtA) which is thought to protect against Zn cellular toxicity; however, not without a reduction in cell viability above 22 μm (zinc was measured at 42 mM in zinc-treated soils here). As could be expected, the five distinct chloroplast sub-OTUs within the five Eukaryota OTUs: Eudicot, Monocot, Bryophyta, Bryophyta-Pottiaceae, and Bacillariophyceae, all clustered as most similar to cyanobacteria when all 552 soil DA OTUs and isolated species spikes were compared (corresponding mitochondrial sub-OTUs were also positioned closest to Alphaproteobacteria; **Supplementary File 4**).

#### Chloroflexi

Bacteria within the Chloroflexi phylum were highly abundant in the soil and, with one exception, were less abundant in zinc-, copper-, and three metal-treated soil when compared to control or lead treatment. The exception within the phylum was the OTU Sphaerobacteraceae, which was more abundant in soils contaminated with all three metals than when compared to control, increasing by 2.13 log_2_FC (**Figure 4C**). Uncharacterized Caldilineaceae was reduced in abundance in three metal-treated soil when compared to control and lead treatments, by -2.08 and -2.25 log_2_FC, respectively. The *Dehalococcoides* OTU was reduced in abundance in three metal-treated soil when compared to all other treatments, ranging from -1.52 to -1.89 log_2_FC, and was also the most abundant genus or species taxonomic level OTU in the phylum, with 1.54 and 1.55% of total normalized counts in control and lead-treated soil, respectively. The uncharacterized Chloroflexi OTU was very highly abundant in control and lead-treated soils, 5.23 and 4.34% total normalized counts, respectively. It is interesting to note that the highly abundant sub-OTU within uncharacterized Chloroflexi were not similar (ID < 90%, **Supplementary File 3**) to any Chloroflexi species that have been isolated and sequenced to date, with the most similar isolated species being *Longilinea arvoryzae*, an anaerobic, mesophilic bacteria whose growth is enhanced when co-cultured with hydrogenotrophic methanogens (potentially of interest when considered alongside the parallel reduction in abundance of potential archaeal hydrogenotrophic methanogens). The first species to be identified in *Dehalococcoides* was *D. ethenogenes*, a noted bioremediator which is capable of degrading common halogenated hydrocarbon pollutants (Yamada et al., 2007), such as PCBs (polychlorinated biphenyls), but has less established tolerance of heavy metals. Sphaerobacteraceae comprised only a single sub-OTU, the most similar isolated species (ID 90%) was *Sphaerobacter thermophilus*, unsurprising, as the only species in the genus. The generalized intolerance to high metal content is documented in Chloroflexi (such as to copper; Berg et al., 2012a) but this tolerance is less well characterized, although perhaps could be expected in Sphaerobacteraceae as *S. thermophilus* was isolated from municipal sludge, commonly high in metal content (Hensel et al., 1989) (since reclassified to Chloroflexi).

#### Firmicutes

The *Carboxydibrachium* and *Thermoanaerobacter* OTUs were both significantly and substantially reduced in abundance upon treatment with three metals and zinc when compared to control, lead, and copper treatments ranging from -2.40 to -3.81 log_2_FC (**Figures 4A,C,D,F,G,I**). This pattern of response was in contrast to the *Lactococcus raffinolactis* OTU which was significantly more abundant (1.52 log_2_FC) in soils treated with three metals when compared to control. Each of the three Firmicutes OTUs, when unpacked, was comprised of only a single sub-OTU. The *Carboxydibrachium* and *Thermoanaerobacter* OTUs were highly distinctive from the closest cultured or isolated species; the most similar 16S rRNA partial gene sequence being the Chloroflexi bacteria *L. arvoryzae* (91% blastn identity) and *Desulfoplanes formicivorans* (81% identity blastn identity) respectively. Again, contrary to this, *L. raffinolactis* was an ambiguous amplicon which did not vary (100% identity) between the *Lactococcus* species *L. chungangensis*, *L. laudensis*, or *L. raffinolactis* (**Supplementary File 3**). Looijesteijn et al. (2001) quite elegantly demonstrated how extracellular polysaccharides, considered an essential component of metal sorption in bacteria (Gupta and Diwan, 2016), of the closely related *Lactococcus lactis* (94% blastn identity to the *L. raffinolactis* OTU) conferred copper tolerance. The specific strains of *L. raffinolactis* (DSM 20443 16S) and *L. chungangensis* (CAU 28 16S) were both isolated from activated sludge foam, likely to have high heavy metal content, from Cheonan wastewater plant in South Korea (Cho et al., 2008).

#### Planctomycetes

Planctomycetes are a fascinating group of bacteria which are distinct from the classical prokaryotic phenotype in containing, for example, cell internal compartmentalization including a membrane bound nucleoid (Fuerst, 1995; Fuerst and Sagulenko, 2011). The phylum contained only a single differentially abundant OTU, which could only be characterized at the order taxonomic level of Planctomycetales, and was significantly reduced in abundance upon zinc and three metal treatments when compared to control, lead-, or copper-treated soil, ranging from -0.83 to -0.91 log_2_FC (**Figures 4A,C,D,F,G,I**). Unpacking the OTU showed 35 highly diverse sequences had been grouped, the highest similarity to known 16S rRNA gene sequence from isolated Planctomycetales species was with the sub-OTU Planctomycetales sequence 27, sharing 95% identity to *Zavarzinella formosa* and Planctomycetales sequence 24 and 31 sharing approximately 93% identity with *Pirellula staleyi* (**Supplementary File 3**). Other sequences had <90% similarity to isolated or cultured species but were most similar to *Algisphaera agarilytica*, *Blastopirellula cremea*, *Blastopirellula marina*, *Bythopirellula goksoyri*, *Gemmata obscuriglobus*, *Planctomyces brasiliensis*, *Planctomyces limnophilus*, *Roseimaritima ulvae*, as well as the Nitrospirae *Thermodesulfovibrio hydrogeniphilus*, the Actinobacteria *Streptomyces burgazadensis*, and the Deltaproteobacteria *Desulfuromonas michiganensis*. While these findings are interesting in clearly suggesting a quite wide array of unknown Planctomycetales species exist in healthy soil at the Shanghai field site, they are impossible to resolve given our current limited knowledge base within the phylum. Susceptibility to long-term copper exposure has been seen in Planctomycetes previously (Berg et al., 2012b) although studies have been limited to phylum level assessment.

#### Latescibacteria

Latescibacteria is a recent proposed candidate phylum (previously WS3) born from *in silico* analysis using rRNA gene as an identification marker and single-cell amplified genomics (Youssef et al., 2015). Member species are seemingly ubiquitous within the environment but a representative species has yet to be cultured (Farag et al., 2017; **Supplementary File 3**, no isolated spikes from phylum). The Latescibacteria OTU abundance was significantly reduced, but not abolished, in soil treated with three metals when compared with all other treatments, ranging from -1.21 log_2_FC compared to zinc to -1.92 log_2_FC compared to control soil (**Figures 4C,F,H,I**). While the metabolism and diversity of ecological function of many Latescibacteria species are understandably still rather cryptic (Farag et al., 2017), there is evidence of member tolerance to organic anthropogenic pollutants (Hernandez-Raquet et al., 2006), the susceptibility to metal suggested here, however, has previously been observed by Berg et al. (2012b) who observed a decrease in relative abundance of Latescibacteria due to copper pollution.

#### Actinobacteria

**FIGURE 6:**
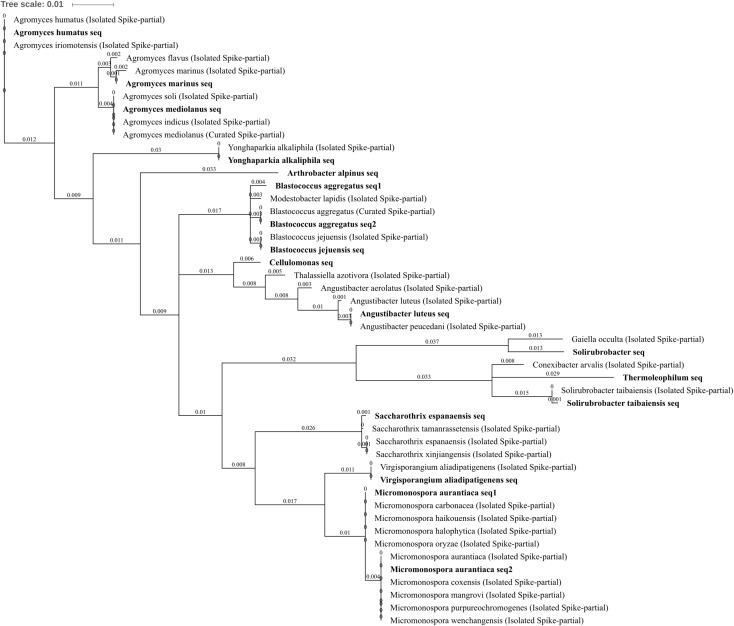
Actinobacteria phylogenetic tree. Jukes–Cantor model with neighbor-joining and >50% support threshold (1,000 replications). Sub-OTUs (distinct amplicons) are highlighted in bold with spike (into the tree) sequences included as the most similar hits from the NCBI bacteria and archaea 16S rRNA gene database. All accession numbers and sequences (sub-OTUs and tree spikes) are provided in **Supplementary File 2**. The value “0.01” on the scale bar represents 1 substitution in 100 bp.

Actinobacteria are ubiquitous through all environments including all extreme environments currently known to harbor microorganisms (Bull, 2011). The amplified community belonging to Actinobacteria did not have a uniform response to metal treatment across all 15 constituent OTUs. *Arthrobacter alpinus*, a psychrophilic species (able to grow at extremely cold temperatures), decreased in abundance in soils treated with zinc, copper, or three metals when compared to untreated soil by -2.73, -2.66, and -2.55 log_2_FC, respectively (**Figures 4A–C**). Similarly, *Virgisporangium aliadipatigenens* significantly decreased when three metals were applied to soil compared to lead-treated soil, -2.91 log_2_FC, whereas *Saccharothrix espanaensis* OTU abundance was entirely abolished from soil treated with all three metals. *Yonghaparkia alkaliphila* was significantly increased in abundance in soil treated with all three metals when compared against all other treatments and, along with *Angustibacter luteus*, was absent from control and lead-treated soil. *Blastococcus aggregatus* and *Blastococcus jejuensis* both increased in abundance in three metal-treated soil when compared to control or lead treatment, ranging from 2.07 to 3.14 log_2_FC. An unknown but close relative of *Y. alkaliphila* (96% identity) was one of the 199 bacteria successfully isolated from uranium mine tailings in the Athabasca Basin, Canada, and subsequently determined to be one of only 15 dual-metal hyper-tolerant species capable of growing on media with high arsenic and selenium concentrations (Bondici et al., 2013). The family Geodermatophilaceae, harboring the genus *Blastococcus*, is also recognized as containing broadly metal tolerant bacterial species with the genome of members such as *Blastococcus saxobsidens* containing putative metal tolerance determinants (Sghaier et al., 2016). Three *Agromyces* sp. OTUs were identified, *A. marinus*, *A. mediolanus*, and *A. humatus. A. marinus* and *A. mediolanus* were significantly reduced in abundance due to three metal treatment, *A. marinus* when compared to control treatment (-2.55 log_2_FC) and *A. mediolanus* when compared to lead or zinc treatment (-2.66 and -2.73 log_2_FC). The more distinct of the three (**Figure 6**), *A. humatus*, significantly increased and was unique to three metal-treated soil. Each of these *Agromyces* species OTUs containing only a single sub-OTU and are an interesting example of the value of differentiating species using the 16S rRNA gene as an identification technology if possible, or the potential risk of not doing so, as fundamentally distinct responses to stress or treatment may occur within the biological system between species of the same genus. There is evidence of an *Agromyces* species able to tolerate high metal concentrations (6 mM zinc), such as that isolated from soil of a lead/zinc mine (Corretto et al., 2016), *Agromyces aureus*, which is more similar to the metal tolerant *Agromyces* species identified here (98% identity to *A. humatus*).

Within the class Thermoleophilia, the *Solirubrobacter* OTU (which was very highly abundant in the soil) was in higher abundance across in zinc, copper, and three metals when compared to control treatment, by 2.26, 2.01, and 3.26 log_2_FC, as well as in higher abundance in zinc and three metals when compared to lead, by 1.91 and 2.91 log_2_FC, respectively (**Figures 4A–D,F**). This was in contrast to the *Solirubrobacter taibaiensis* OTU and the similar (94% identical sites) *Thermoleophilum* OTU, which had reduced abundance in soil treated with three metals compared to control (-2.36 log_2_FC) and lead (-2.55 log_2_FC), or just lead (-1.52 log_2_FC) treatment, respectively. Unpacking of the OTU (**Figure 6**) revealed only a single sub-OTU within each bin and that the *S. taibaiensis* OTU and *Solirubrobacter* OTU are distinct (88% identical sites) with *S. taibaiensis* sharing 100% similarity with the isolated species whereas the closest isolated species to the *Solirubrobacter* OTU was *Gaiella occulta* (96% identity). Given the very broad metal tolerance indicated by the *Solirubrobacter* OTU, it is unsurprising that the closely related *Gaiella* sp. have been previously been identified by Xu et al. (2017) as increasing in relative abundance in zinc and lead polluted mining sites compared to control (low metal content) regions in Yunnan province, China. The *Micromonospora aurantiaca* OTU was increased in zinc-treated soil when compared to either control or three metal treatment, 3.75 and 3.41 log_2_FC, respectively. The amplified region was ambiguous for a number of *Micromonospora* species. The *M. aurantiaca* OTU could be separated into two amplicons (sub-OTUs) which varied only by a single nucleotide (position 319), which distinguishes the amplified 16s rRNA gene region submitted from the isolated species *M. oryzae*, *M. halophytica*, *M. haikouensis*, and *M. carbonacea* from *M. aurantiaca*, *M. coxensis*, *M. mangrovi*, *M. purpureochromogenes*, and *M. wenchangensis*. While *Micromonospora* species, such as *M. aurantiaca* (Hamdali et al., 2008) and *M. nickelidurans* (Lin et al., 2015), have been isolated from high metal content soils, this potentially specific tolerance to zinc has not been documented. As the genus is known to contain plant growth promoting bacteria (Tekaya et al., 2012), such as *M. carbonacea* (El-Tarabily et al., 2000), this increase in abundance may be indicative of secondary interactions with tolerant plant species.

#### Proteobacteria

Similar to Actinobacteria, Beta-, Delta-, and Gammaproteobacteria did not have a uniform response to metal treatment with some member species significantly increasing and some significantly decreasing in abundance when the soil was treated with one or all three metals.

##### Alphaproteobacteria

All the identified differentially abundant OTUs in Alphaproteobacteria were in higher abundance in zinc, copper, and/or three metal treatment when compared to control or Pb-treated soil (**Figures 4A–F**). Two OTUs could be annotated only at the level of order, uncharacterized Caulobacterales and uncharacterized Rhodospirillales, both of which comprised only a single high abundance sub-OTU each (**Supplementary File 2**). At the genus and species level, *Phenylobacterium*, *Rhodobacter*, *Erythrobacter*, *Porphyrobacter*, *Sphingomonas*, *Novosphingobium naphthalenivorans*, *Pseudolabrys taiwanensis*, and *Reyranella massiliensis* OTUs were all identified as differentially abundant due to treatment.

The *Phenylobacterium* OTU was increased in abundance in three metal treatment when compared to control soil by 1.39 log_2_FC. The OTU comprised eight highly similar sub-OTUs, all of which varied in a non-random manner across the amplicon (variation limited to specific regions across multiple sub-OTUs makes sequencing error an unlikely cause for even minor amplicon variation). The most similar *Phenylobacterium* species which have been isolated were *P. muchangponense*, *P. immobile*, and *P. mobile*. The *Rhodobacter* OTU increased in abundance in zinc-treated soil when compared to control or lead soils as well as when treated with all three metals when compared against lead-treated soil (**Figures 4A,B,F**). *Rhodobacter* was comprised of five sub-OTUs, four of which were within 96% identity of one another (also varying in a non-random fashion at the same amplicon positions: 56–60, 100, and 112–119 nt) and similar to isolated *Tabrizicola aquatica* and *Catellibacterium nectariphilum*, whereas *Rhodobacter* seq4 was highly dissimilar to the other sub-OTUs (sharing 86% identical sites with *Rhodobacter* seq1) and more closely related to *Skermanella stibiiresistens* (**Supplementary File 3**). The *Erythrobacter* OTU was in higher abundance in three metal treatment and absent from control soil, whereas the *Porphyrobacter* OTU significantly increased in abundance in three metal-treated soil when compared to control, lead, zinc, or copper conditions, by 3.72, 2.61, 2.39, and 2.74 log_2_FC, respectively (**Figures 4C,F,G,I**). Both *Erythrobacter* and *Porphyrobacter* reside within the family Erythrobacteraceae, aerobic and pleomorphic bacteria (able to alter size and shape in response to environmental conditions). The closest isolated species to both of the most abundant sequences (sub-OTUs) in the *Erythrobacter* and *Porphyrobacter* OTUs belonged to the *Altererythrobacter* genus (also within Erythrobacteraceae), being *Altererythrobacter troitsensis* and *Altererythrobacter dongtanensis. Altererythrobacter* species have been observed to grow in liquid media containing high heavy metal concentrations, such as *Altererythrobacter atlanticus* (Wu et al., 2014) and *A. dongtanensis* was first isolated from the Dongtan Wetland, China, a region rich in lead, zinc, and copper (Quan et al., 2010), suggesting high general metal tolerance may be present in multiple species across the family.

The *Sphingomonas* OTU was significantly increased in abundance after three metal treatment when compared to control, lead, and copper soils (**Figures 4C,F,I**), by 2.17, 1.80, and 1.32 log_2_FC, respectively, and was comprised of 17 sub-OTUs. This high number of sub-OTUs could represent a generally high 16S rRNA gene copy number in species of the genera (although limited research has been conducted, only single copies have been found in two *Sphingomonas* species; Fegatella et al., 1998; Tiirola et al., 2002) or the presence of a number of different species of the genera in the ecosystem increasing in abundance due to increased metal concentration. *Sphingomonas* species are commonly found in contaminated soil, albeit those containing high levels of polycyclic aromatic hydrocarbon in particular (due to their capacity to utilize these compounds as a carbon source). The 17 sub-OTUs sequences shared 87% identical sites and were similar to isolated species from Korea: *Sphingomonas flava* (Gyeonggi Province; Du et al., 2015), *S. daechungensis*, *S. sediminicola* (Daecheong Dam; An et al., 2013; Huy et al., 2014), *S. oryziterrae* (Jinju Province; Chung et al., 2011), and *S. vulcanisoli* (Gotjawal Forest; Lee et al., 2015) as well as Japan: *S. jaspsi* Tottori (Asker et al., 2007) (**Supplementary File 3**).

Alphaproteobacteria, were identified as the dominant enriching phylum in a study of Chinese soil contaminated with crude oil conducted by Yang et al. (2014). This trial, and others (Lan et al., 2011; Liao et al., 2015; Rodgers-Vieira et al., 2015), highlight the Alphaproteobacteria community as comprising *Phenylobacterium* species, which have the (eponymous) rare preference to metabolize phenyl moieties from heterocyclic compounds as a carbon source (most sugars and amino acids are not used) (Eberspächer, 2015) as well as *Sphingomonas* and *Novosphingobium* species as having important potential as petroleum hydrocarbon degraders in Chinese soils. However, in terms of thriving within a metal contaminated environment, *Phenylobacterium*, *Sphingobium*, *Novosphingobium* as well as, more broadly, the orders of *Caulobacterales* and *Rhodospirillales*, are common within the communities of heavy metal rich soil [examples include soil from a copper mines in China (Li et al., 2013) and Brazil (Pereira et al., 2015), alongside Bacteroidetes species also identified here (i.e., *Ohtaekwangia*) in metal contaminated soil (Cáliz et al., 2013)]. Three species level OTUs were positively identified as differentially abundant, *N. naphthalenivorans*, *P. taiwanensis*, and *R. massiliensis. N. naphthalenivorans* increased in three metal-treated soils (being absent from control soil; **Figure 4C**) and was comprised of two similar sub-OTUs (96.9% identical sites), one of which was also highly similar to the sequence of isolated *Novosphingobium stygium. P. taiwanensis*, which comprised only a single sub-OTU, increased in abundance in three metal treatment and was entirely absent from either control or lead-treated soil. *R. massiliensis* increased in three metal treatment and was absent in control, lead-, and copper-treated soils (**Figures 4C,F,I**, low and/or poorly distributed counts in zinc-treated soil). Huang et al. (2011) identified *P. taiwanensis* within extremely polluted Pb–Zn mine tailings in Guangdong Province, China, strongly indicating high tolerance metal tolerance in species (zinc levels were 28,428 mg kg^-1^). *R. massiliensis* has been observed as decreasing in abundance with increasing degrees of metal pollution (Hong et al., 2015) (which included relatively high chromium and cadmium levels); however, recent research has detected the bacteria from the genus in the biofilm community of copper and zinc contaminated drinking water (Buse et al., 2017), suggesting specific tolerance to these metals (a lack of generalized metal tolerance).

##### Betaproteobacteria

**FIGURE 7:**
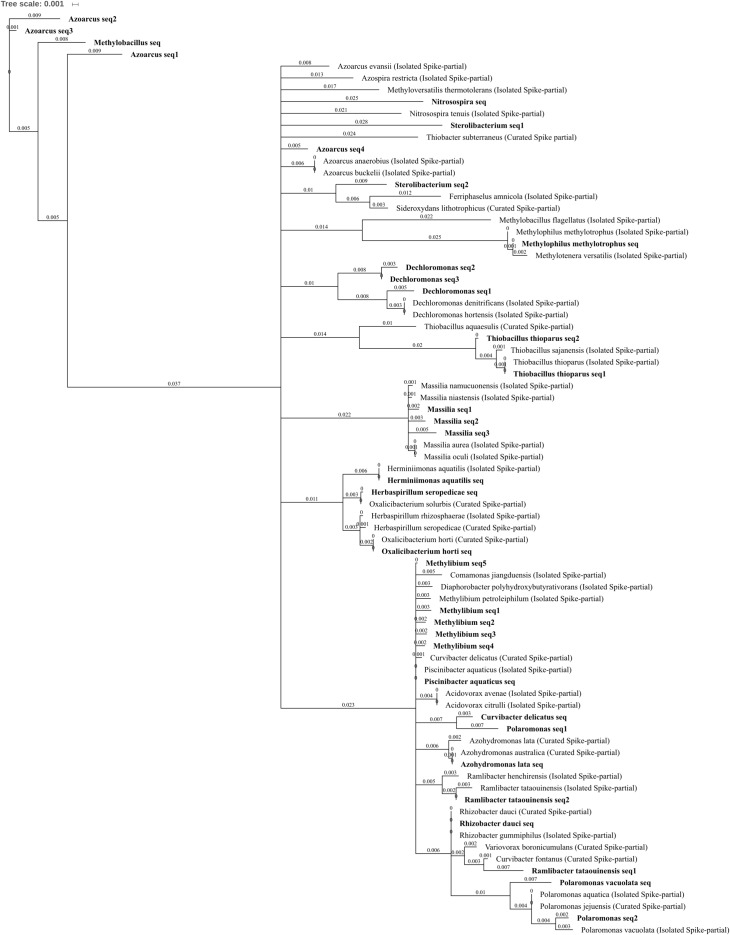
Betaproteobacteria phylogenetic tree. Jukes–Cantor model with neighbor-joining and >50% support threshold (1,000 replications). Sub-OTUs (distinct amplicons) are highlighted in bold with spike (into the tree) sequences included as the most similar hits from the NCBI bacteria and archaea 16S rRNA gene database. All accession numbers and sequences (sub-OTUs and tree spikes) are provided in **Supplementary File 2**. The value “0.001” on the scale bar represents 1 substitution in 1,000 bp.

Four OTUs from the Order Burkholderiales were reduced or abolished in abundance by three metal treatment when compared to control and/or lead-treated soil, comprising the species *Azohydromonas lata* as well as OTUs which are currently poorly characterized at the family level (**Figure 7**) but are part of the *Methylibium* monophyletic group: *Methylibium*, *Piscinibacter aquaticus*, and *Rhizobacter dauci*, previously placed in Gammaproteobacteria (Stackebrandt et al., 2009). The type *Methylibium* species is *M. petroleiphilum*, when the isolated *M. petroleiphilum* was compared to the five sub-OTUs comprising the OTU identified here *Methylibium* seq2 shared 99.2% identical sites. *M. petroleiphilum* has a well-known capacity to degrade petroleum hydrocarbons and was isolated from oil refinery biofilters in methyl *tert*-butyl ether rich culture (Nakatsu et al., 2006). As such, it is unsurprising that aromatic and alkane degradation-associated genes were identified in the sequenced *M. petroleiphilum* PM1 genome alongside recognized metal tolerance genes (Kane et al., 2007), making this reduction in abundance in metal polluted soil unexpected.

A total of 15 OTUs in Betaproteobacteria were significantly increased in abundance due to zinc, copper, and/or three metal treatment when compared to control or lead-treated soil, spanning the families Comamonadaceae, Oxalobacteraceae, Methylophilaceae, Nitrosomonadaceae, and Rhodocyclaceae. These included seven genera level OTUs (*Polaromonas*, *Massilia*, *Methylobacillus*, *Nitrosospira*, *Azoarcus*, *Dechloromonas*, and *Sterolibacterium*) and seven species level OTUs (*Oxalicibacterium horti*, *Thiobacillus thioparus*, *Methylophilus methylotrophus*, *Polaromonas vacuolata*, *Ramlibacter tataouinensis*, *Herbaspirillum seropedicae*, and *Herminiimonas aquatilis*). Metal treatment significantly increased *P. vacuolata* and *Nitrosospira* OTUs, which were both absent in control soils, while *O. horti*, *M. methylotrophus*, *H. seropedicae*, and *H. aquatilis* were all absent from both control and lead-treated soils (**Figures 4C,F**). The phylum also comprised some of the most abundant OTUs within metal-treated soil; *Methylobacillus* had extremely high relative abundance of 0.7, 1.4, and 2.5% total normalized counts in zinc-, copper-, and three metal-treated soil, respectively whereas *Azoarcus* had 1.3% total normalized counts in both copper- and three metal-treated soil, respectively. When differentiating the sequences comprising the *Azoarcus* OTU, the most abundant three sub-OTUs (*Azoarcus* seq1–3) shared <90% identity with any isolated or curated species within the NCBI 16S bacterial and archaea database (**Figure 7**) while the least abundant (*Azoarcus* seq4) was highly similar to the isolated species *A. buckelii* and *A. anaerobius. Azoarcus* species are well-known nitrogen-fixing, aromatic compound degraders often found in anthropogenically contaminated wastewater (Rabus et al., 2014). The high tolerance to metal suggested by these findings is supported by the genome sequence of *Azoarcus* sp. strain CIB (Martín-Moldes et al., 2015), which identified heavy metal resistance gene clusters. *P. vacuolata* and *R. tataouinensis* (first isolated from meteorite fragments buried in the desert near Tataouine, Tunisia) are both extremophiles being extremely psychrophilic(Irgens et al., 1996) and desiccation tolerant (De Luca et al., 2011), respectively. *Nitrosospira* has previously been identified as the only ammonia oxidizer retrieved from the extremely heavy metal rich Idaho Coeur d’Alene River (United States) (Rastogi et al., 2011). The arrival of *Nitrosospira* in three metal-treated soil is interesting given the important role of the genus in nitrification and in light of the decrease in ammonia-oxidizing *Nitrososphaera* and parallel increase in nitrite-oxidizing Nitrospirae in three metal-treated soil.

##### Deltaproteobacteria

Only four OTUs were identified as differentially abundant due to metal treatment, uncharacterized Deltaproteobacteria, uncharacterized Desulfuromonadales, uncharacterized Geobacteraceae, and *Haliangium*. The majority of the amplicons where poorly characterized so were predominately binned within the broad uncharacterized Deltaproteobacteria OTU, which was reduced in abundance by -0.96 log_2_FC by three metal treatment when compared to lead-treated soil, or uncharacterized Desulfuromonadales, which increased in abundance due to three metal and zinc treatments when compared to control soil by 1.23 and 1.12 log_2_FC, respectively (**Figures 4A,C**). The breadth of variation in sequences within these OTUs mean the utility of a shift in abundance is quite limited (most likely representing only a “net” change in a group of unknown species). The uncharacterized Deltaproteobacteria OTU was comprised of 27 distinct sub-OTUs whereas the uncharacterized Desulfuromonadales contained nine sub-OTUs with the most abundant five representative sequences (**Supplementary File 3**) having no similar isolated species within >90% sequence identity (within the NCBI bacteria and archaea 16S rRNA gene database). Uncharacterized Geobacteraceae was reduced in abundance by the three metal treatment compared to lead-treated soil, by -2.60 log_2_FC whereas *Haliangium* was increased in abundance in three metal soils by 2.47 and 1.80 log_2_FC compared to control and lead-treated soils, respectively (**Figures 4C,F**). The uncharacterized Geobacteraceae OTU comprised only two very similar sub-OTUs, the most abundant (uncharacterized Geobacteraceae seq1) was most similar to the isolated species *Geobacter argillaceus*, interestingly a dissimilatory Fe(III) reducing bacteria (Shelobolina et al., 2007). *Haliangium* comprised only a single sub-OTU and the most similar isolated species (sharing only 91.9% identical sites) was *Haliangium ochraceum*, a halophilic myxobacterium which has previously been identified in copper, zinc, and lead rich soil (Gillan et al., 2017).

##### Gammaproteobacteria

**FIGURE 8:**
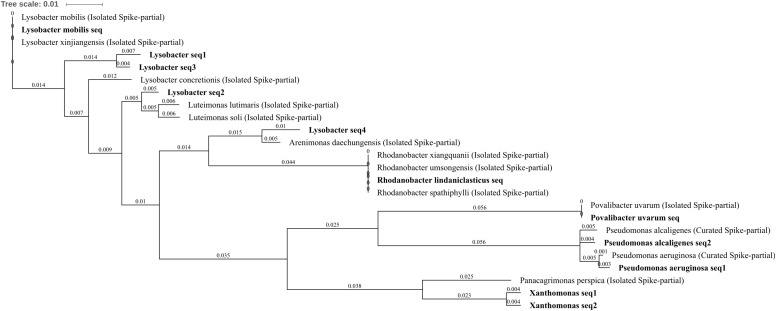
Gammaproteobacteria phylogenetic tree. Jukes–Cantor model with neighbor-joining and >50% support threshold (1,000 replications). Sub-OTUs (distinct amplicons) are highlighted in bold with spike (into the tree) sequences included as the most similar hits from the NCBI bacteria and archaea 16S rRNA gene database. All accession numbers and sequences (sub-OTUs and tree spikes) are provided in **Supplementary File 2**. The value “0.01” on the scale bar represents 1 substitution in 100 bp.

Differentially abundant Gammaproteobacteria were well characterized with seven OTUs identified *Povalibacter uvarum*, *Pseudomonas aeruginosa*, *Pseudomonas alcaligenes*, *Rhodanobacter lindaniclasticus*, *Lysobacter*, *Lysobacter mobilis*, and *Xanthomonas. P. aeruginosa* was reduced in abundance within three metal- or zinc-treated soils when compared to control or lead by between -1.86 and -2.45 log_2_FC, suggesting a potential susceptibility to the impact of zinc within the soil, whereas the *P. alcaligenes* OTU was abolished by three metal treatment (**Figures 4A,C,D,F**). The OTUs *R. lindaniclasticus*, *Lysobacter*, *L. mobilis* were all significantly increased in three metal-treated soil. *Lysobacter*, in particular, was abundant in three metal-treated soil (1.0% total normalized counts) as well as substantially increased compared to control, lead, and zinc treatments by 3.58, 3.16, and, 2.83 log_2_FC, respectively. *L. mobilis* was absent from control and lead-treated soils while *R. lindaniclasticus* was unique to three metal-treated soil. The *Xanthomonas* OTU was increased in abundance in copper treatment when compared to zinc-treated soils (1.94 log_2_FC) and comprising two very similar sub-OTUs with the most similar isolated species in the NCBI 16S bacteria and archaea database being *Panacagrimonas perspica. R. lindaniclasticus* comprised only a single sub-OTU which could not distinguish between the isolated *Rhodanobacter* species *R. xiangquanii*, *R. umsongensis*, and *R. spathiphylli* (**Figure 8**). This was similar to the *L. mobilis* OTU which also comprised only a single sub-OTU which could not distinguish isolated *Lysobacter* species *L. mobilis* and *L. xinjiangensis*. Heavy metal tolerance has been observed in a number of *Lysobacter* species and the tolerance of *L. mobilis* suggested by these results is perhaps unsurprising as it was first isolated from an abandoned lead–zinc mine in Guangdong Province, China (Yang et al., 2015). In *Rhodanobacter*, the genes of zinc resistance associated operons are known to be highly mobile (LGT) between species which are frequently present in metal contaminated groundwater (Hemme et al., 2016).

#### Nitrospirae

The species within the phylum Nitrospirae are somewhat understudied given their widespread presence throughout the environment and their important role in nitrification, commonly being the predominant nitrite-oxidizing bacteria (NOB) in wastewater treatment plants (Daims et al., 2001; Lücker et al., 2010). The *Nitrospira moscoviensis* OTU was significantly increased in abundance when soil was treated with all three metals when compared to control (2.97 log_2_FC; **Figure 4C**). This is fascinating in light of the increased abundance in the ammonia-oxidizing *Nitrosospira* (see Betaproteobacteria), which has previously been found alongside nitrite-oxidizing *Nitrospira* species in heavy metal rich wastewater systems (Schramm et al., 1998; Dionisi et al., 2002). Further to this, only a single sequence cluster was present (<1% sequence variation) when the OTU was unpacked, which was positively identified (no known species ambiguity for this amplicon) as the *N. moscoviensis* isolated from corroded iron pipes in Russia (Ehrich et al., 1995), suggesting metal tolerance is potentially a hallmark of the species. When considered alongside the underlying functional role in nitrification (as a nitrite oxidizer), it is likely this species is a (metal) contamination specific occupant of this ecological niche. Such community modification may go some way in helping to understand the previously observed, unexpected variation in nitrification rates within lead and copper contaminated soils (Sauvé et al., 1999).

#### Acidobacteria and Chlamydiae

The Acidobacteria and Chlamydiae phyla both contain a single differentially abundant OTU, the family level annotated uncharacterized Acidobacteriaceae and genus level *Parachlamydia*. Uncharacterized Acidobacteriaceae was increased in both three metal- and copper-treated soils when compared to control by 0.87 and 1.04 log_2_FC (**Figures 4B,C**). The OTU contained 14 sub-OTUs, all of which were dissimilar to well characterized species, sharing less than 85% identity with any isolated species present in the NCBI bacteria and archaea 16S database. *Parachlamydia* was significantly increased in three metal treatment and absent from control, lead and copper containing soils (low abundance and confidence in zinc). The *Parachlamydia* OTU contained only a single sub-OTU which was most similar to two isolated *Simkania negevensis* and *Parachlamydia acanthamoebae* (sharing ∼90% identity) implying the potential for a specific amoebae tolerance niche in diversely metal polluted soil.

#### Bacteroidetes

**FIGURE 9:**
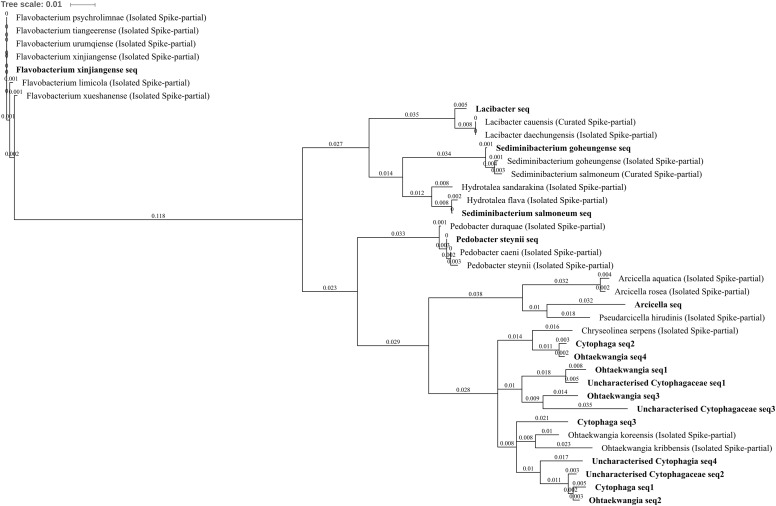
Bacteroidetes phylogenetic tree. Jukes–Cantor model with neighbor-joining and >50% support threshold (1,000 replications). Sub-OTUs (distinct amplicons) are highlighted in bold with spike (into the tree) sequences included as the most similar hits from the NCBI bacteria and archaea 16S rRNA gene database. All accession numbers and sequences (sub-OTUs and tree spikes) are provided in **Supplementary File 2**. The value “0.01” on the scale bar represents 1 substitution in 100 bp.

All of the differentially abundant OTUs identified from the phylum Bacteroidetes were increased in zinc-, copper-, and/or three metal-treated soil with respect to control and/or lead soils. These comprised uncharacterized Cytophagia, uncharacterized Cytophagaceae, *Cytophaga*, *Lacibacter*, *Arcicella*, *Ohtaekwangia*, *Flavobacterium xinjiangense*, *Pedobacter steynii*, *Sediminibacterium goheungense*, and *Sediminibacterium salmoneum*. Chitinophagaceae, *Lacibacter*, and *S. goheungense* OTUs were significantly increased in abundance within three metal treatment compared to control and lead-treated soil whereas *S. salmoneum* and *Arcicella* were unique to three metal-treated soils (**Figures 4C,F,H,I**). Each of these OTUs was made up of only a single sub-OTU. The *Lacibacter* OTU was highly similar to 16S rRNA gene sequence from isolated *Lacibacter cauensis* and *Lacibacter daechungensis* (which could not be distinguished using this amplicon) whereas the *S. salmoneum* was similar (99% identical sites) to the isolated *Hydrotalea flava* (**Figure 9**). The *P. steynii* OTU was increased in abundance in three metal treatment and absent from control and lead-treated soils, and also contained only a single sub-OTU similar (>99% identical sites) to both *P. steynii* and *Pedobacter caeni. F. xinjiangense* was increased in abundance in three metal treatment when compared to control, lead-, and copper-treated soils (4.31, 4.25, and 2.78 log_2_FC, respectively) as well as in zinc treatment compared to control and lead-treated soils (3.35 and 3.29 log_2_FC, respectively). The OTU comprised only a single sub-OTU which was identical with the isolated *F. xinjiangense* but also shared 100% coverage and identity (blastn) with *Flavobacterium urumqiense*, *Flavobacterium psychrolimnae*, and *Flavobacterium tiangeerense*, but not other *Flavobacterium* species (the genus comprises >100 species; Piotrowska-Seget et al., 2005) (**Figure 9**). This is an interesting example where, from a technical standpoint, it would be incorrect to automate the selection of OTU classification to either the genus or the species level, demonstrating the necessity to observe potential annotation on an amplicon by amplicon basis and to allow for natural biological variation to be considered when assessing the utility of a 16S rRNA gene fragment as a marker for microbial identification.

The *Ohtaekwangia* OTU was in higher abundance in each of zinc-, copper-, and three metal-treated soils when compared to control, by 2.56, 3.30, and 3.80 log_2_FC, respectively, as well as in three metal soil when compared to lead-treated soil by 2.55 log_2_FC (**Figures 4A–C,F**). The *Ohtaekwangia* OTU was also extremely abundant in zinc-, copper-, and three metal-treated soils, comprising 0.6, 1.0, and 1.5% total normalized counts in each respective treatment. The *Ohtaekwangia* genus was only been discovered relatively recently and currently comprises only two distinct species *O. koreensis* and *O. kribbensis.* The *O. koreensis* strain 3B-2^T^ and *O. kribbensis* strain 10AO^T^ are considered to have at least one 16S rRNA gene sequence of 97.5% similarity; although for their 16S rRNA gene fragment aligning with the 515F–907R amplicon used here, they share only 95.9% similarity. Both were isolated using low-nutrient media (Yoon et al., 2011) from coast sand collected from Saemangeum (western Korean peninsula). While *Ohtaekwangia* have yet to be studied exhaustively, they are currently being explored for their unique secondary metabolism including the production pyrroloquinolines (the novel marinoquinolines B–F were isolated from the genus; Okanya et al., 2011). The biological function of these compounds is currently cryptic, although some antibacterial and antifungal activity is recognized and investigations toward antimalarial utility are ongoing, so roles in abiotic stress tolerance are possible. The identification of *Ohtaekwangia* OTU in other challenging environments is common, with examples including: the most abundant OTU in a wastewater treatment plant in Seoul, Korea (Kim et al., 2014), highly increased in soil after 6 years of heavy metal (Cr, Cu, Zn, and Pb) and polycyclic aromatic hydrocarbons contamination in France (Bourceret et al., 2016), drainage from a copper mine in Brazil (Pereira et al., 2015), silver nanoparticle contaminated soil (McGee et al., 2017) and from all biofilm communities in a pilot-scale constructed wetland treating industrial wastewater from Suzhou City, China (Xu et al., 2016). *Ohtaekwangia* OTU were also identified in fascinating research performed by Feder et al. (2015) as part of a natural biofilm community whose presence substantially increased Cu removal (∼40–60%) by a gravel filter within an urban drainage system.

## Conclusion

While significant shifts in this amplified community are seen to be relatively common within phyla, the broader picture is that this amplified community was surprisingly resilient to change by high levels of anthropomorphic pollution. This could be due to robust resilience of individual species within the community, such as from *Methylobacillus*, *Solirubrobacter*, and *Ohtaekwangia* which were highly abundant in metal-treated soils, or the novel or replacement functionality brought into the community by genera and/or species specifically adapted to high zinc and copper concentration conditions (which were not detected in control soils), such as the highly abundant *P. vacuolata*, *Y. alkaliphila*, and *Nitrosospira*. To reveal the new functionality enabling tolerance of high metal concentrations within the amplified community, future studies should concentrate on *in situ* metatranscriptomic investigations using high tolerance, differentially abundant species as a guide to help navigate what will undoubtedly be an extremely complex background of gene expression. The relative decreases in *Nitrososphaera* (ammonia-oxidizing archaea) and increases in tolerant *Nitrosospira* and Nitrospirae (ammonia and NOB) in metal contaminated soils is a useful hint that such studies may find it rewarding to extend functional inquiry beyond a single kingdom. While preventing and reducing environmental pollution is essential, in particular with regards to the potential lasting health impact of persistent pollutants, it is reassuring that the magnitude and complexity of natural variation does seemingly bestow a high capacity for some sections of the biosphere to resist the consequences of human activity upon the environmental.

## Author Contributions

GV and ML designed the study. SK and GV performed the experiments. All authors analyzed the data and contributed to the manuscript.

## Conflict of Interest Statement

The authors declare that the research was conducted in the absence of any commercial or financial relationships that could be construed as a potential conflict of interest.

## Supplementary Material

**FILE 1 |** Additional bioinformatics data.

**FILE 2 |** All differentially abundance OTU, sub-OTU and known sequence spikes including normalized counts and annotation.

**FILE 3 |** Phylogenetic trees: Cyanobacteria, Chloroflexi, Firmicutes, Planctomycetes, Latescibacteria, Actinobacteria, Proteobacteria (Alpha and Delta), Nitrospirae, Acidobacteria and Chlamydiae.

**FILE 4 |** Phylogenetic tree/all. All sequences compared (no support threshold applied). All sequence information has been made available through the European Nucleotide Archive. Accession: PRJEB23241. ID: ena-STUDY-MCGILL UNIVERSITY AND GENOME QUEBEC INNOVATION CEN-27-10-2017-22:13:00:673-828
