# Supplementary material for: The Response of a 16S Ribosomal RNA Gene Fragment Amplified Community to Lead, Zinc, and Copper Pollution in a Shanghai Field Trial
Source: Front Microbiol. 2018 Mar 1;9:366. doi: 10.3389/fmicb.2018.00366 (PMC5838024; doi:10.3389/fmicb.2018.00366)
Supplement: Supplementary file 1 [file Data_Sheet_1.DOCX]

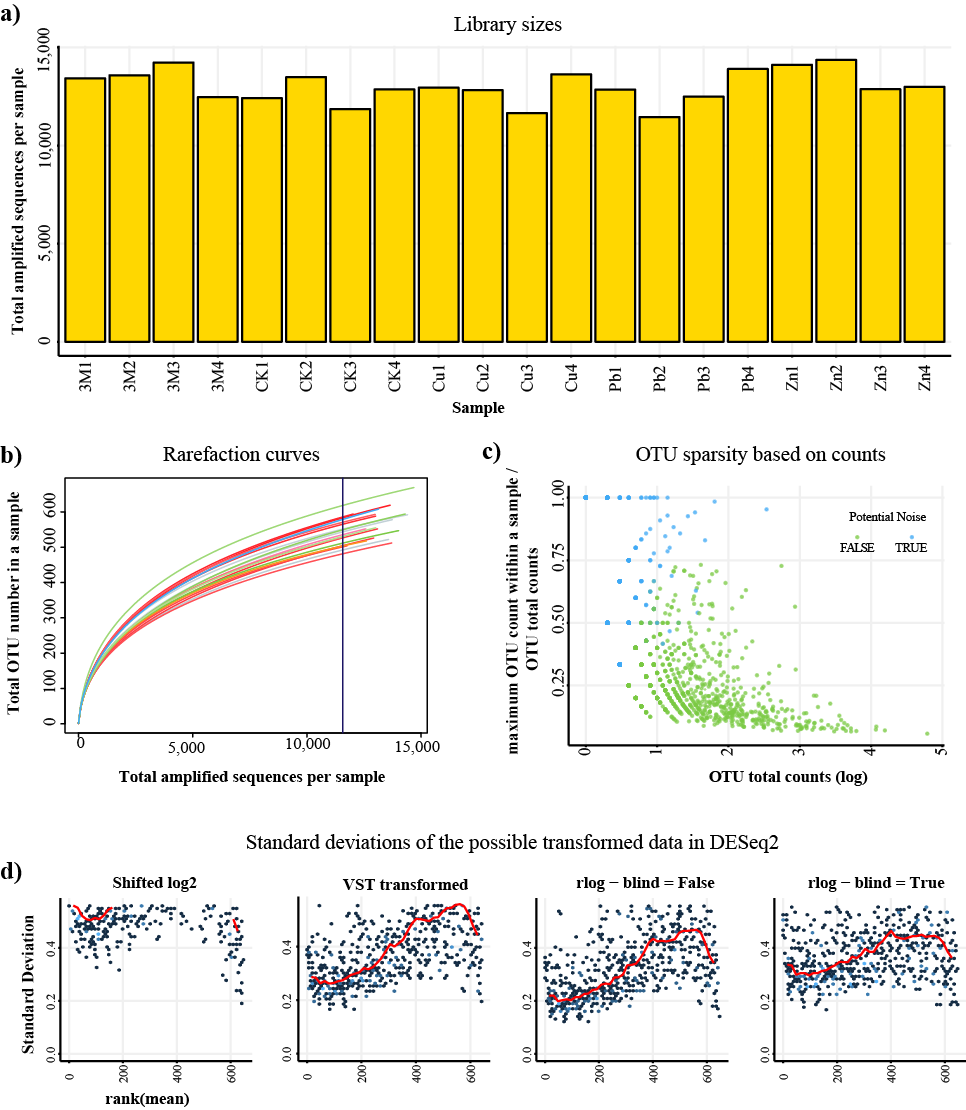


**a)** Total number of sequences per samples as utilized in the input for DESeq2 program. **b)** OTU rarefaction analysis on all samples before differential abundance analysis **c)** Representation of the sparsity defined as the row sum of counts and the proportion of count within a single sample. **d)** Representation of the effect of four different transformations on the variance across samples, using the shifted logarithm transformation, the variance stabilizing transformation, the regularized log transformation (blind = FLASE) and the regularized log transformation (blind = TRUE). The latter has been used in this experiment.

| **Alpha diversity**  **Comparison** | **Index** | **estimate.mean in group1** | **estimate.mean in group2** | **p.value** | **statistic.t** | **conf.int1** | **conf.int2** |
| --- | --- | --- | --- | --- | --- | --- | --- |
| 3M_vs_control | Chao1 | 797.6163 | 811.8118 | 0.6910 | -0.4180 | -98.0873 | 69.6964 |
| 3M_vs_control | Fisher | 121.3128 | 127.3226 | 0.3571 | -1.0071 | -21.0411 | 9.0214 |
| 3M_vs_control | InvSimpson | 16.7874 | 13.9862 | 0.2087 | 1.5069 | -2.4310 | 8.0333 |
| 3M_vs_control | Observed | 573.0000 | 588.2500 | 0.5223 | -0.6815 | -70.7578 | 40.2578 |
| 3M_vs_control | se.chao1 | 42.4453 | 41.4818 | 0.7974 | 0.2709 | -8.2237 | 10.1507 |
| 3M_vs_control | Shannon | 4.1834 | 4.1120 | 0.4566 | 0.8097 | -0.1587 | 0.3015 |
| 3M_vs_control | Simpson | 0.9385 | 0.9281 | 0.1966 | 1.5166 | -0.0080 | 0.0289 |
| 3M_vs_Copper | Chao1 | 797.6163 | 765.1280 | 0.4182 | 0.8692 | -58.9754 | 123.9520 |
| 3M_vs_Copper | Fisher | 121.3128 | 109.7169 | 0.0956 | 2.0971 | -3.0550 | 26.2468 |
| 3M_vs_Copper | InvSimpson | 16.7874 | 14.4589 | 0.2739 | 1.3118 | -3.0594 | 7.7163 |
| 3M_vs_Copper | Observed | 573.0000 | 523.5000 | 0.0616 | 2.4137 | -3.5273 | 102.5273 |
| 3M_vs_Copper | se.chao1 | 42.4453 | 47.2631 | 0.2926 | -1.1538 | -15.0457 | 5.4102 |
| 3M_vs_Copper | Shannon | 4.1834 | 4.0372 | 0.1517 | 1.8874 | -0.0946 | 0.3869 |
| 3M_vs_Copper | Simpson | 0.9385 | 0.9307 | 0.2974 | 1.2206 | -0.0110 | 0.0267 |
| 3M_vs_Lead | Chao1 | 797.6163 | 759.4917 | 0.3517 | 1.0095 | -54.3085 | 130.5578 |
| 3M_vs_Lead | Fisher | 121.3128 | 116.0765 | 0.4474 | 0.8141 | -10.5964 | 21.0690 |
| 3M_vs_Lead | InvSimpson | 16.7874 | 13.1942 | 0.1276 | 1.9471 | -1.6547 | 8.8410 |
| 3M_vs_Lead | Observed | 573.0000 | 546.5000 | 0.3158 | 1.0946 | -32.7839 | 85.7839 |
| 3M_vs_Lead | se.chao1 | 42.4453 | 42.0689 | 0.9198 | 0.1060 | -8.8070 | 9.5598 |
| 3M_vs_Lead | Shannon | 4.1834 | 4.0298 | 0.1381 | 1.8388 | -0.0762 | 0.3835 |
| 3M_vs_Lead | Simpson | 0.9385 | 0.9237 | 0.0946 | 2.0645 | -0.0037 | 0.0335 |
| 3M_vs_Zinc | Chao1 | 797.6163 | 888.7493 | 0.0400 | -2.6283 | -176.4358 | -5.8301 |
| 3M_vs_Zinc | Fisher | 121.3128 | 129.7760 | 0.3026 | -1.1297 | -26.8908 | 9.9643 |
| 3M_vs_Zinc | InvSimpson | 16.7874 | 16.1309 | 0.8012 | 0.2632 | -5.4476 | 6.7605 |
| 3M_vs_Zinc | Observed | 573.0000 | 606.5000 | 0.3026 | -1.1361 | -107.1214 | 40.1214 |
| 3M_vs_Zinc | se.chao1 | 42.4453 | 51.8536 | 0.1404 | -1.7338 | -23.1371 | 4.3206 |
| 3M_vs_Zinc | Shannon | 4.1834 | 4.2006 | 0.9022 | -0.1288 | -0.3541 | 0.3197 |
| 3M_vs_Zinc | Simpson | 0.9385 | 0.9358 | 0.7737 | 0.3009 | -0.0193 | 0.0247 |
| Copper_vs_control | Chao1 | 765.1280 | 811.8118 | 0.2231 | -1.3656 | -131.2242 | 37.8566 |
| Copper_vs_control | Fisher | 109.7169 | 127.3226 | 0.0073 | -4.1126 | -28.2756 | -6.9359 |
| Copper_vs_control | InvSimpson | 14.4589 | 13.9862 | 0.5648 | 0.6186 | -1.5280 | 2.4734 |
| Copper_vs_control | Observed | 523.5000 | 588.2500 | 0.0111 | -3.7261 | -108.0080 | -21.4920 |
| Copper_vs_control | se.chao1 | 47.2631 | 41.4818 | 0.1477 | 1.7044 | -2.8798 | 14.4424 |
| Copper_vs_control | Shannon | 4.0372 | 4.1120 | 0.1853 | -1.6571 | -0.2092 | 0.0597 |
| Copper_vs_control | Simpson | 0.9307 | 0.9281 | 0.5020 | 0.7249 | -0.0068 | 0.0121 |
| Copper_vs_Lead | Chao1 | 765.1280 | 759.4917 | 0.8869 | 0.1484 | -87.2829 | 98.5556 |
| Copper_vs_Lead | Fisher | 109.7169 | 116.0765 | 0.2516 | -1.2959 | -18.9775 | 6.2583 |
| Copper_vs_Lead | InvSimpson | 14.4589 | 13.1942 | 0.1454 | 1.7311 | -0.6265 | 3.1559 |
| Copper_vs_Lead | Observed | 523.5000 | 546.5000 | 0.2945 | -1.1687 | -73.3708 | 27.3708 |
| Copper_vs_Lead | se.chao1 | 47.2631 | 42.0689 | 0.1846 | 1.5337 | -3.4616 | 13.8498 |
| Copper_vs_Lead | Shannon | 4.0372 | 4.0298 | 0.8433 | 0.2121 | -0.0937 | 0.1086 |
| Copper_vs_Lead | Simpson | 0.9307 | 0.9237 | 0.1603 | 1.6883 | -0.0041 | 0.0182 |
| Copper_vs_Zinc | Chao1 | 765.1280 | 888.7493 | 0.0127 | -3.5426 | -209.5399 | -37.7027 |
| Copper_vs_Zinc | Fisher | 109.7169 | 129.7760 | 0.0303 | -3.2188 | -37.0596 | -3.0587 |
| Copper_vs_Zinc | InvSimpson | 14.4589 | 16.1309 | 0.4233 | -0.9138 | -7.2410 | 3.8969 |
| Copper_vs_Zinc | Observed | 523.5000 | 606.5000 | 0.0311 | -3.2050 | -153.9275 | -12.0725 |
| Copper_vs_Zinc | se.chao1 | 47.2631 | 51.8536 | 0.4270 | -0.8629 | -18.1983 | 9.0172 |
| Copper_vs_Zinc | Shannon | 4.0372 | 4.2006 | 0.2330 | -1.4824 | -0.5101 | 0.1833 |
| Copper_vs_Zinc | Simpson | 0.9307 | 0.9358 | 0.4964 | -0.7584 | -0.0252 | 0.0149 |
| Lead_vs_control | Chao1 | 759.4917 | 811.8118 | 0.1839 | -1.5117 | -138.0858 | 33.4455 |
| Lead_vs_control | Fisher | 116.0765 | 127.3226 | 0.0841 | -2.0840 | -24.5767 | 2.0844 |
| Lead_vs_control | InvSimpson | 13.1942 | 13.9862 | 0.4205 | -0.8648 | -3.0349 | 1.4509 |
| Lead_vs_control | Observed | 546.5000 | 588.2500 | 0.1033 | -1.9310 | -95.0858 | 11.5858 |
| Lead_vs_control | se.chao1 | 42.0689 | 41.4818 | 0.8278 | 0.2272 | -5.7374 | 6.9116 |
| Lead_vs_control | Shannon | 4.0298 | 4.1120 | 0.1885 | -1.4966 | -0.2189 | 0.0545 |
| Lead_vs_control | Simpson | 0.9237 | 0.9281 | 0.4056 | -0.8962 | -0.0164 | 0.0076 |
| Zinc_vs_control | Chao1 | 888.7493 | 811.8118 | 0.0489 | 2.4655 | 0.5339 | 153.3411 |
| Zinc_vs_control | Fisher | 129.7760 | 127.3226 | 0.7265 | 0.3704 | -14.6526 | 19.5594 |
| Zinc_vs_control | InvSimpson | 16.1309 | 13.9862 | 0.3274 | 1.1221 | -3.2648 | 7.5543 |
| Zinc_vs_control | Observed | 606.5000 | 588.2500 | 0.5358 | 0.6660 | -52.9188 | 89.4188 |
| Zinc_vs_control | se.chao1 | 51.8536 | 41.4818 | 0.0997 | 2.1389 | -3.1285 | 23.8721 |
| Zinc_vs_control | Shannon | 4.2006 | 4.1120 | 0.4951 | 0.7511 | -0.2414 | 0.4187 |
| Zinc_vs_control | Simpson | 0.9358 | 0.9281 | 0.3389 | 1.0744 | -0.0118 | 0.0274 |
| Zinc_vs_Lead | Chao1 | 888.7493 | 759.4917 | 0.0112 | 3.6606 | 42.1741 | 216.3412 |
| Zinc_vs_Lead | Fisher | 129.7760 | 116.0765 | 0.1041 | 1.9446 | -3.9146 | 31.3136 |
| Zinc_vs_Lead | InvSimpson | 16.1309 | 13.1942 | 0.2019 | 1.5471 | -2.4897 | 8.3631 |
| Zinc_vs_Lead | Observed | 606.5000 | 546.5000 | 0.0887 | 2.0749 | -12.7730 | 132.7730 |
| Zinc_vs_Lead | se.chao1 | 51.8536 | 42.0689 | 0.1142 | 2.0193 | -3.7186 | 23.2879 |
| Zinc_vs_Lead | Shannon | 4.2006 | 4.0298 | 0.2189 | 1.4910 | -0.1639 | 0.5056 |
| Zinc_vs_Lead | Simpson | 0.9358 | 0.9237 | 0.1696 | 1.6173 | -0.0075 | 0.0318 |
