# Supplementary material for: The Response of a 16S Ribosomal RNA Gene Fragment Amplified Community to Lead, Zinc, and Copper Pollution in a Shanghai Field Trial
Source: Front Microbiol. 2018 Mar 1;9:366. doi: 10.3389/fmicb.2018.00366 (PMC5838024; doi:10.3389/fmicb.2018.00366)

Acidobacteria
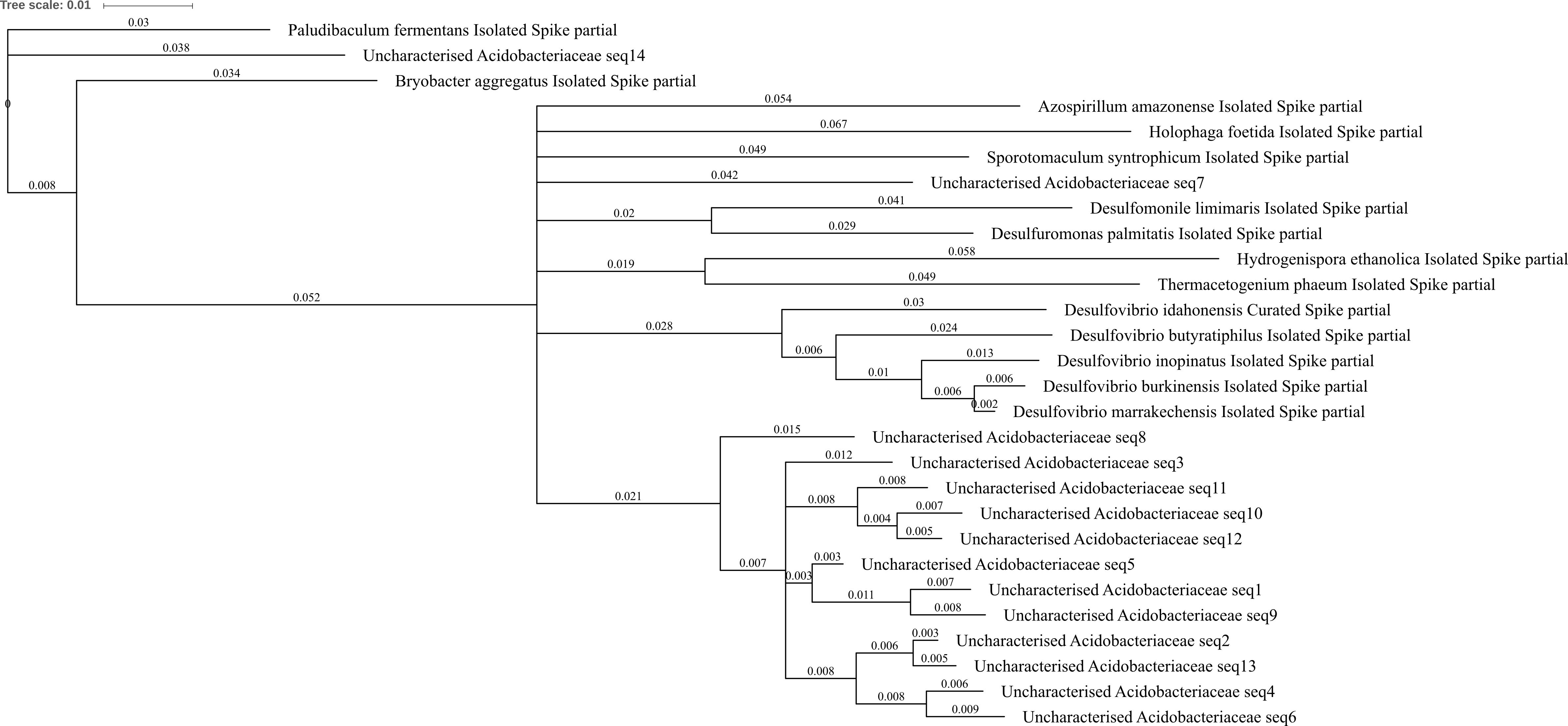


Alphaproteobacteria
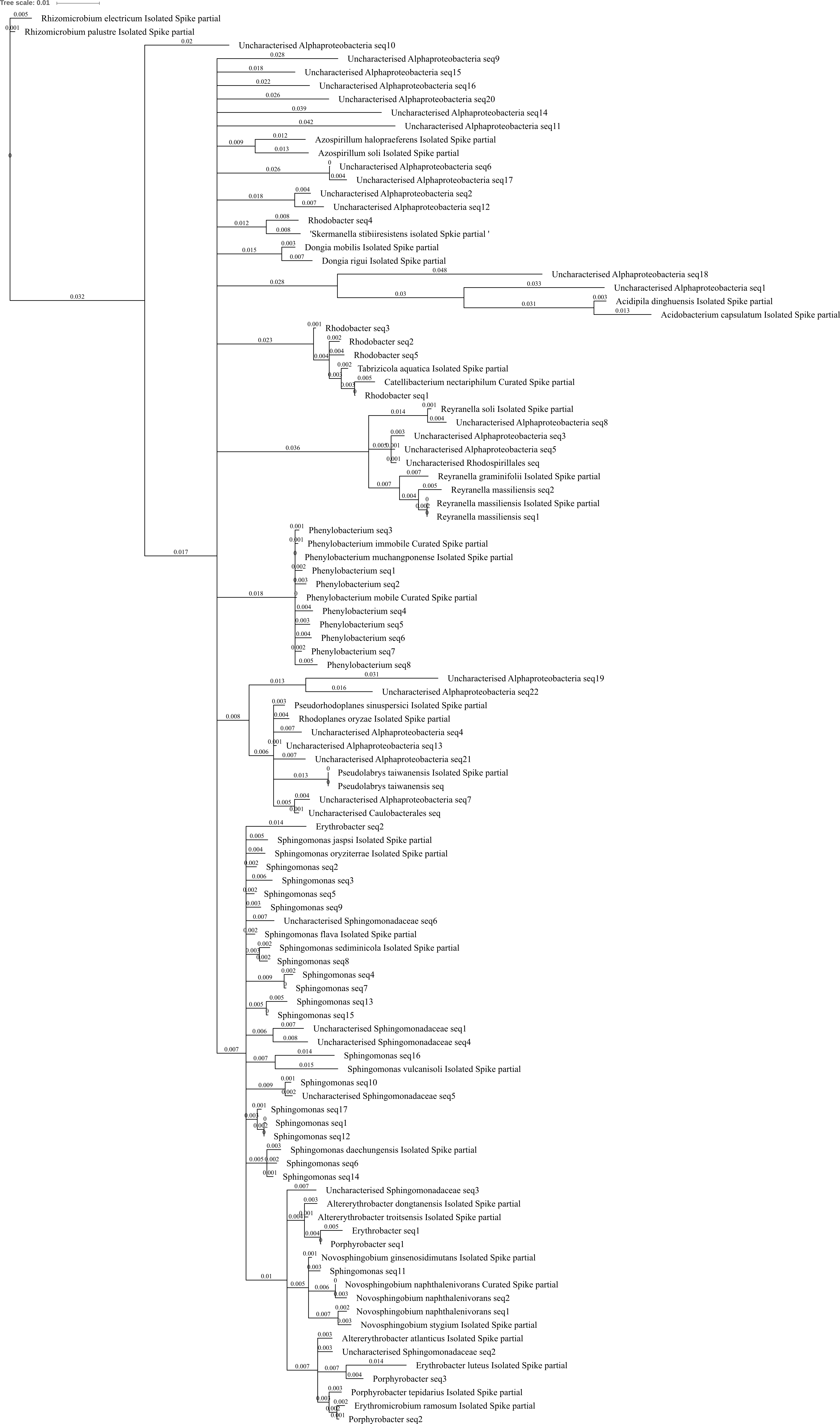


Chlamydiae
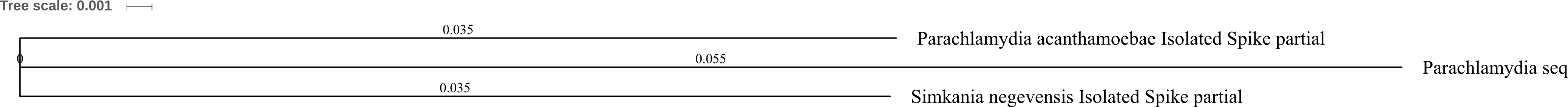
Chloroflexi
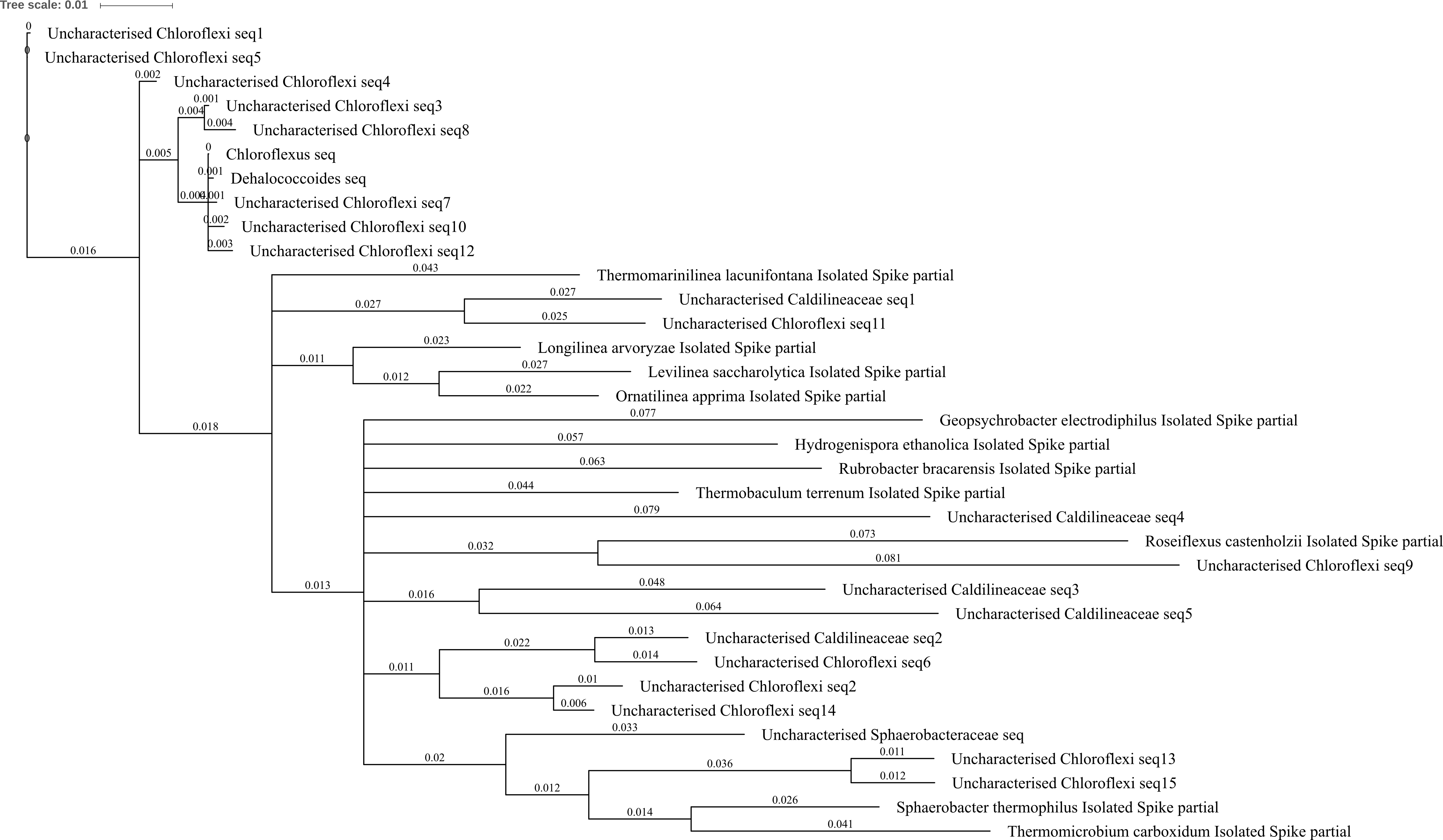
Cyanobacteria
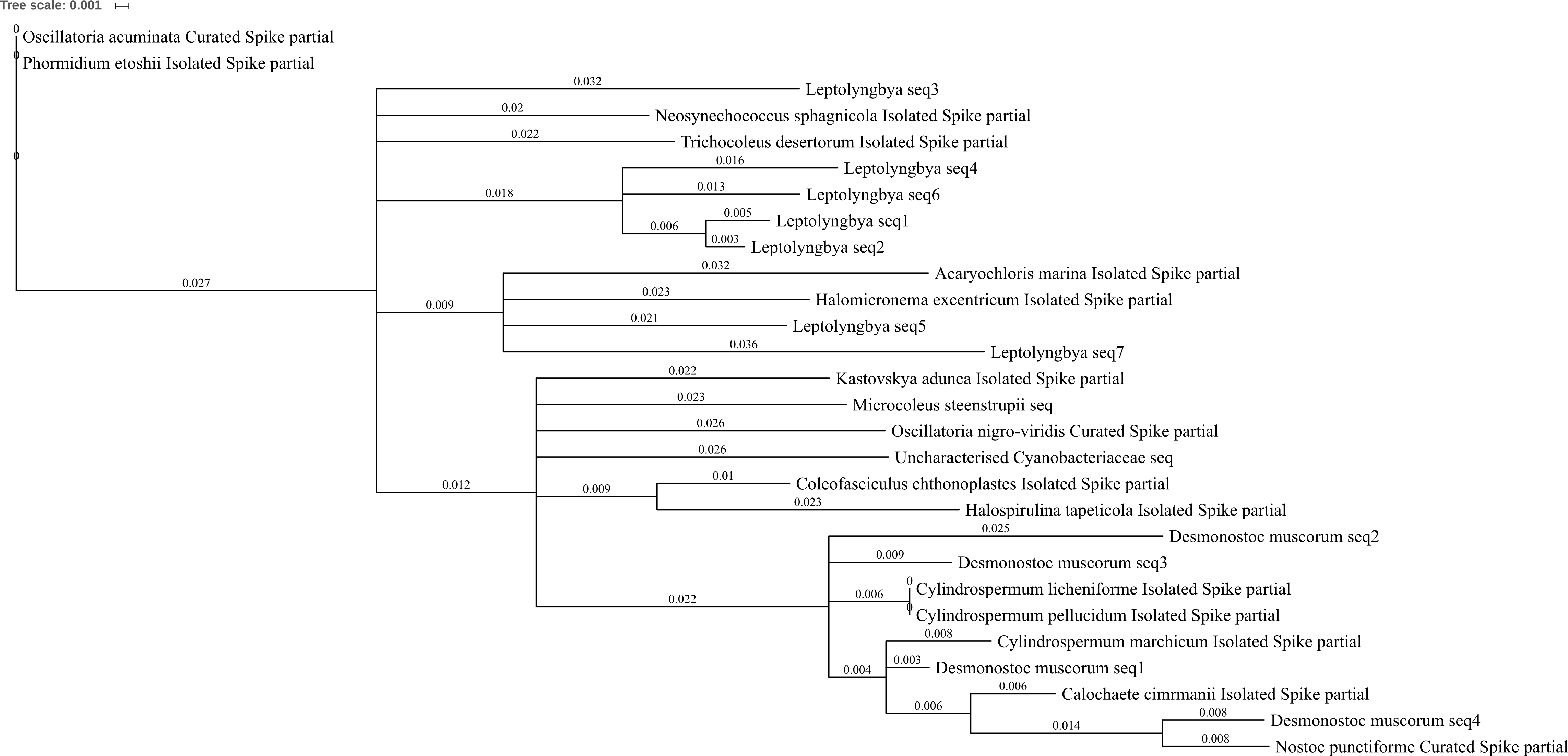


Delatproteobacteria
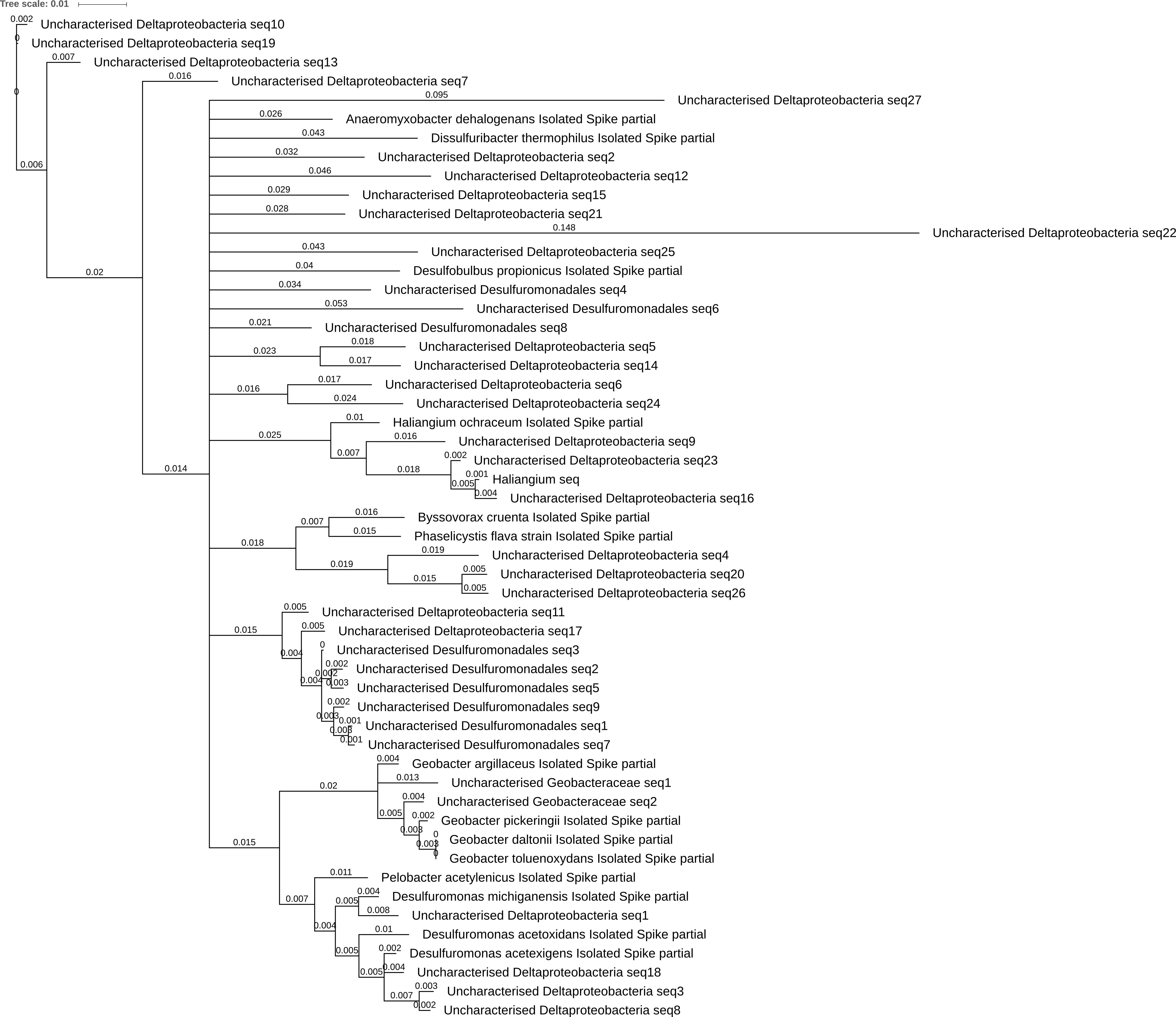
Firmicutes
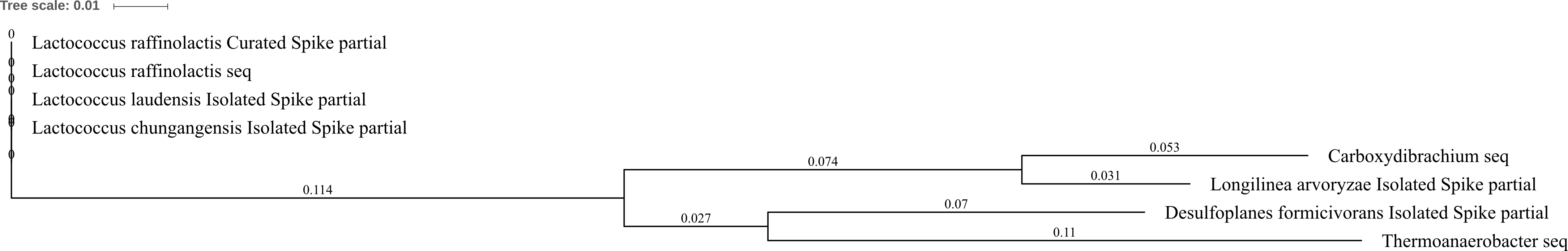
Latescibacteria
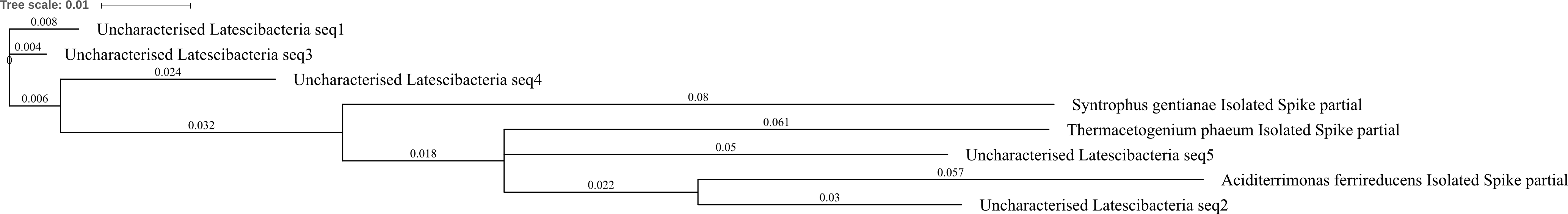


Nitrospoira
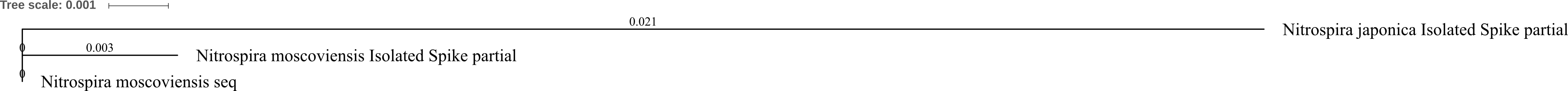
Planctomycetales
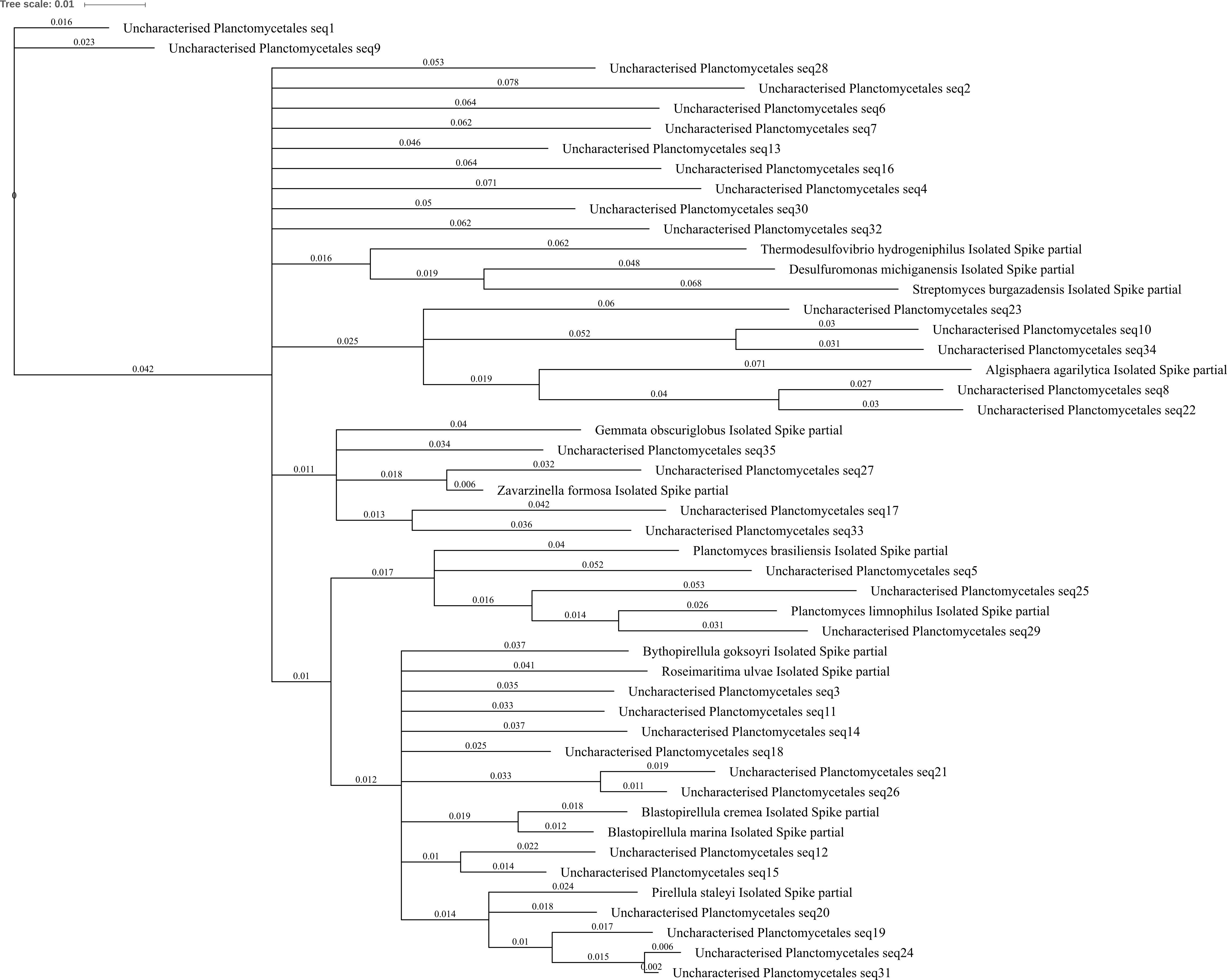

Supplement: Supplementary file 3 [file Data_Sheet_3.DOCX]
